# Heat stress induces specific methylation, transcriptomic and metabolic pattern in dairy cows and their female progeny

**DOI:** 10.1038/s41598-025-01082-3

**Published:** 2025-05-16

**Authors:** Kathrin Halli, Tong Yin, Christian Koch, Stefan Krebs, Sven König

**Affiliations:** 1 Institute of Animal Breeding and Genetics, Justus-Liebig-University, 35390 Giessen, Germany; 2 Educational and Research Centre for Animal Husbandry, Hofgut Neumuehle, 67728 Muenchweiler an der Alsenz, Germany; 3 Gene Center – Laboratory for Functional Genome Analysis, Ludwig-Maximilians-University, 81377 Munich, Germany; 4 Zhejiang Key Laboratory of Dairy Cattle Genetic Improvement and Milk Quality Research, Wenzhou, 32500 People’s Republic of China

**Keywords:** Multi-omics analysis, Dairy cows, Direct heat stress, Time-lagged heat stress, Female progeny, Genetics, Physiology, Environmental sciences

## Abstract

A heat stress (HS) cattle research design was implemented to study HS effects on the three different “omics features” methylations, gene expressions and metabolic pattern from a direct perspective in pregnant cows and from an indirect time-lagged intergenerational perspective in offspring (the respective F1 and as F1 offspring before calving). In this regard, a total number of 88 German Holstein dairy cows and their 93 female calves were blood sampled for DNA and RNA extraction and for metabolic phenotyping, and allocated to HS and respective control groups (the cows (dams) as well as their calves) according to a temperature–humidity threshold of 60. Separate principal component analyses for all “omics-tiers” revealed clear separations of HS from respective control groups, as well as dam—offspring separations according to gene expressions and metabolic pattern. The GO enrichment analyses based on the differentially expressed genes contributed to the detection of 10 significantly overrepresented biological processes in heat stressed dams, and of 95 overrepresented biological processes due to indirect maternal heat stress in calves. With regard to direct HS in dams and the first PCs of the different “omics” features, the correlation coefficient was 0.45 between methylation and gene expression data, 0.62 between expression and metabolites, and 0.38 between methylation and metabolite data. The separation of HS from the control group was very obvious when using the average and weighted average of the first and second components from the three multi-omics datasets. The present study provides extended insights into the complex genetic and physiological mechanisms of HS response in dam and calf groups from different generations, contributing to a deeper understanding of the interplay of prompt and time lagged HS effects between different omics-tiers.

## Introduction

The unequivocal impact of climate change in Central Europe on livestock production has been highlighted in numerous studies. The adverse effects of HS address impaired animal physiology, welfare, health and reproduction. One of the most widely used thermal parameters to assess heat loads in dairy cattle is the temperature-humidity index (THI)^1^, combining the effects of temperature and humidity.

We have recently proposed that direct HS during late gestation, even under German climate conditions, causes altered fat metabolism in dairy cows and calves^2^. In addition, from an across-generation perspective, we found impaired productivity, female fertility and longevity in female progeny due to intrauterine HS (i.e., HS affecting their dams) during late gestation.

Intrauterine HS during late gestation impaired performance traits of F1-offspring^3^, and even induced carry over effects in the following generations^4^. Alterations on postnatal phenotypes might be due to epigenetic modifications during fetal development, such as DNA methylation^5^, histone modifications^6^ or microRNAs^7^, with enhancing or repressing impact on gene expressions^8^. In such context, in utero HS caused altered methylation and gene expressions^9^ when compared to in utero cooled cattle groups. Studies addressing the direct effect of HS during late gestation and during the transition period also reported altered gene expressions^10,11^, and differently expressed mRNAs and miRNAs in blood^12^ and liver^13,14^.

When compared to studies only addressing “single omics types” (e.g., genomics or metabolomics), the integration of multiple omics data types (multi-omics), might contribute to a deeper understanding of the interplay between genomic, physiological and molecular mechanisms^15,16^. To the best of our knowledge, no previous study investigated the effects of direct and maternal HS in dairy cows and their female progeny from a multi-omics and intergenerational approach under German climatic conditions. In consequence, we performed an analysis considering the metabolic and the methylation profiles as well as gene expressions of dairy cows and their female calves suffering from direct or maternal HS during the last eight weeks of gestation and their respective control groups. We hypothesized that different climatic conditions and generations cause differences in genomic and physiological mechanisms, reflected by discrepancies in the metabolic profile, DNA methylations and gene expressions between the animal groups (dams vs. calves and directly or maternally heat stressed animal groups vs. the respective control groups). In this regard, we expected (a) to specify specific DNA methylation and gene expressions between the animal groups; (b) to detect promoters, genes and metabolites significantly determining the grouping of direct or maternal HS; (c) to identify promoters and genes that are significantly associated with the differential gene expressions and metabolite profiles.

## Results

### Principal component analyses

#### Genomic relationships

**Fig. 1:**
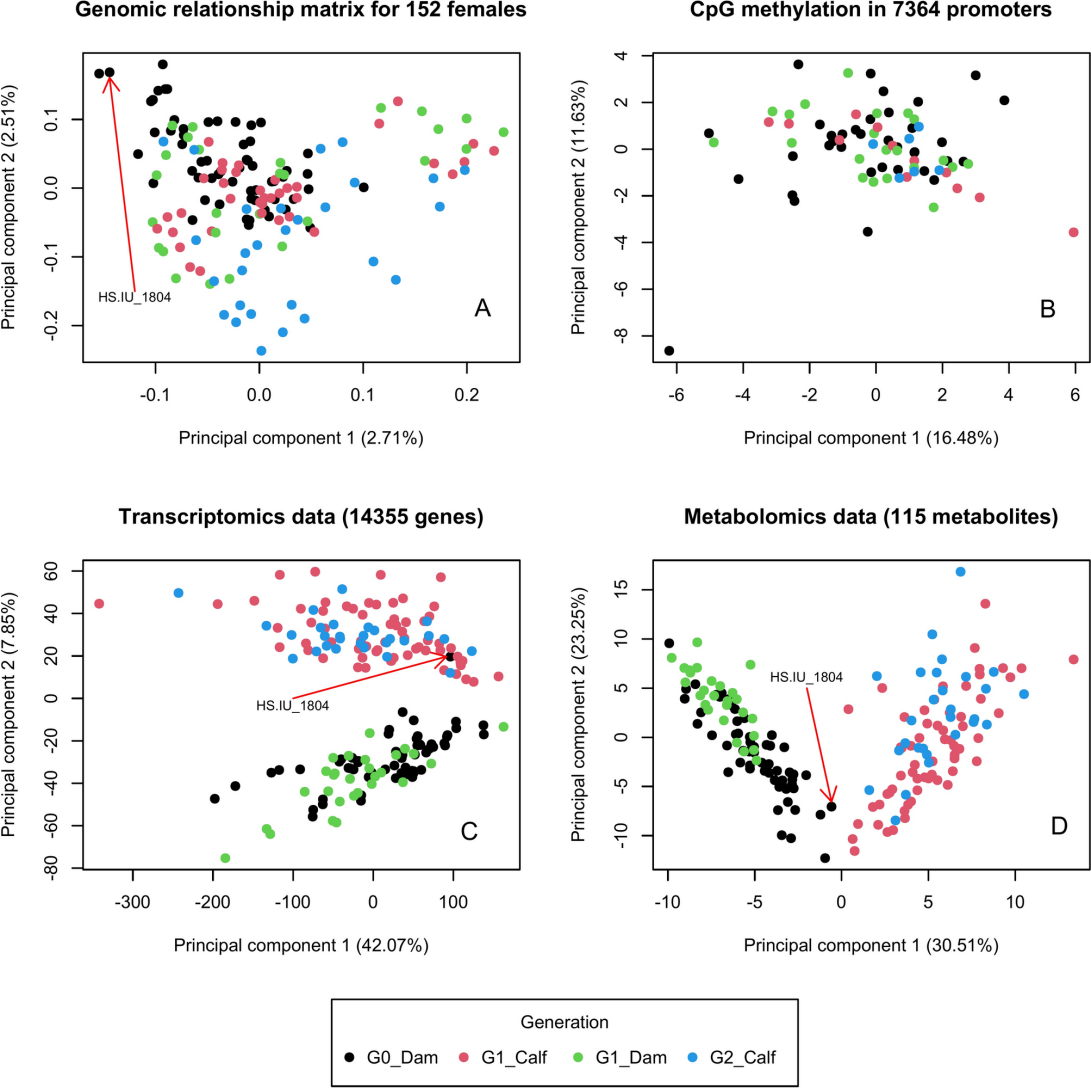
Plot of the first 2 principal components for the genomic relationships (**A**), CPG methylation (**B**), transcriptomic data (**C**) and metabolomics data (**D**) in dams and their calves form different generations G0, G1 and G2.

The genomic relationships between the same animals ranged from 0.95 to 1.57 with an average value of 1.09. For dam–daughter and dam–granddaughter pairs, the average relationships were 0.39 and 0.16, respectively. The first two principal components (PCs) from the principal component analysis (PCA) of the genomic relationship matrix (GRM) are plotted in Fig. 1A, indicating slight separation from the across-generation perspective among dams (F0), F1 animals and F2 calves. The proportion of variance explained by PC1 of genomic relationships was 2.71%. For PC2, the corresponding proportion was 2.51%.

#### Methylation

The first two PCs from the PCA for CpG methylation in 7364 promoters are depicted in Fig. 1B. The animals did not display any distinct clusters when using PCs based on methylation profiles. Nevertheless, the proportion of variance explained by the first two PCs was moderate. The proportion of variance explained by PC1 of CPG methylation was 16.48%. For PC2, the corresponding proportion was 11.63%.

#### Gene expressions

The first two PCs from the PCA for gene expressions (i.e., transcriptomics data from 14355 genes) are depicted in Fig. 1C. In this regard, a clear differentiation between dam and calf groups is very obvious. The proportion of variance explained by PC1 of transcriptomic data was 42.07%. For PC2, the corresponding proportion was 7.85%. The sample (FO dam) HS.IU_1804 was identified as an outlier, as also indicated in the PC plots for the GRM and for metabolomics data. This outlier was excluded from the ongoing analyses.

#### Metabolic profiles

The first two PCs from the PCA for metabolic profiles are plotted in Fig. 1D. Basically, in agreement with the transcriptomic data, metabolic profiles indicate an obvious clustering between dams and calves. The proportion of variance explained by PC1 of metabolomics data was 30.51%. For PC2, the corresponding proportion was 23.25%.

Overall, with regard to the different multi-omics tiers, the first two PCs of methylation, gene expression and metabolic profiles explained larger proportions of variances than the PCs of the GRM.

### Differentially methylated promoters in dams and calves

With regard to the direct HS status in dams, 6 significantly different methylated promoters were identified, including 5 hypermethylated promoters and one hypomethylated promoter. The promoters are located on BTA1, 2, 4, 21, and 27 and in upstream regions of the genes *LSAMP*, *ENSBTAG00000051923*, *GIMAP8*, *ENSBTAG00000051425* and *EBD* (Table S1). The gene *LSAMP* was identified twice, because promoters for two transcripts of the gene were differentially methylated. No biological process was significantly overrepresented in gene ontology (GO) enrichment analyses.

In total, 39 different genes showed differentially methylated promoters with regard to the maternal indirect HS status in calves (Table S1), including 34 protein-coding genes, 5 genes for long non-coding RNAs (lnc_RNA), one gene for small nuclear RNA (snRNA) and one gene for small nucleolar RNA (snoRNA), plus one pseudogene. Transcriptions of three identified genes were initiated by two significant promoters, and only one significant promoter region was detected in the remaining genes. In contrast to the methylation differences in dams, only one promotor was hypermethylated (*AP1B1*) due to indirect HS, and the other promoters indicated declined methylation percentages. The gene *ENSBTAG00000051923* coding lnc_RNA was significantly differently methylated in both HS groups (direct HS dam group and indirect HS calf group) compared to the respective control groups. However, we could not infer significantly overrepresented biological processes and molecular functions via GO enrichment analyses.

### Differentially expressed genes in dams and calves

**Fig. 2:**
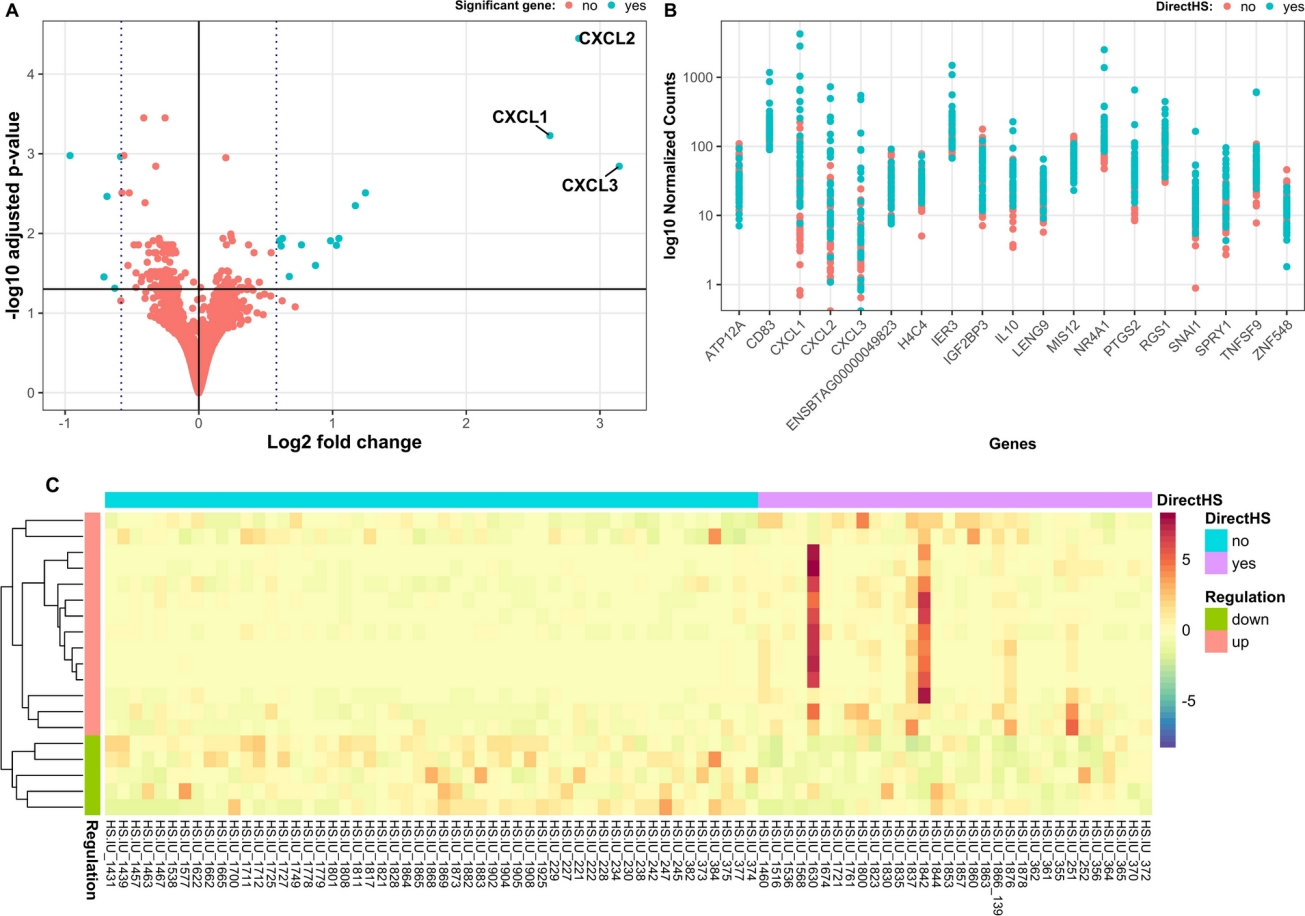
Differentially expressed genes of dams under direct heat stress conditions (turquois dots above the log2 fold change threshold indicate upregulation, turquois dots below the log2 fold change threshold indicate downregulation) (**A**), log10 normalized read counts for gene expressions according to the direct heat stress status of cows (turquois dots indicate heat stress, red dots indicate cows from the control group) (**B**), and contribution of the different samples to up- or downregulation of genes with regard to the direct heat stress status of dams (**C**).

For dams, the analysis of 22,934 genes with nonzero total read counts revealed 383 genes with adjusted *p*-values of expression changes lower than 0.1. Among these genes, 159 genes were upregulated (limit fold change (LFC) > 0), and 224 genes were downregulated (LFC < 0), when comparing the direct HS cow group with the respective control group. The most important differences with regard to gene expressions are outlined in Fig. 2. Number of significantly differentially expressed genes were 19, and only five of them were downregulated (Fig. 2A). However, we did not observe apparent separations of the read counts for the 19 genes between the two groups (direct HS versus control group) (Fig. 2B). Two samples (i.e., HS.IU_1630 and HS.IU_1842) mainly contributed to significantly different expressions of nine genes, and the remaining samples only slightly contributed to differences in gene expressions (Fig. 2C). A considerable proportion of genes, specifically 11,114 (48%), exhibited low expression levels (mean read count < 14). Consequently, these genes were removed before differently gene expression (DGE) analysis.

**Fig. 3:**
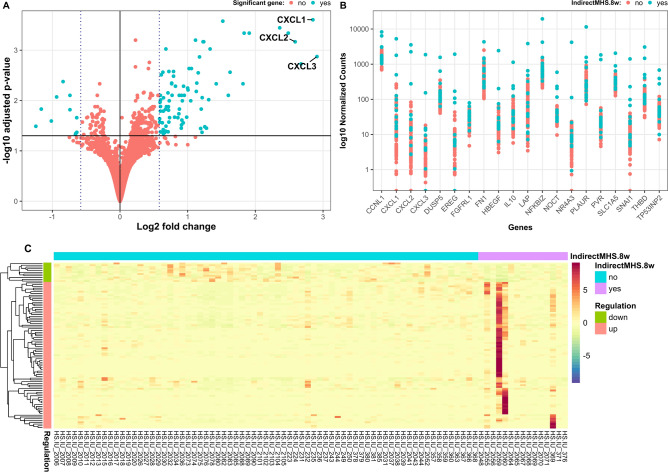
Differentially expressed genes of calves under indirect heat stress conditions (turquois dots above the log2 fold change threshold indicate upregulation, turquois dots below the log2 fold change threshold indicate downregulation) (**A**), log10 normalized read counts for gene expressions according to the indirect heat stress status of calves (turquois dots indicate heat stress, red dots indicate cows from the control group) (**B**), and contribution of the different samples to up- or downregulation of genes with regard to the indirect heat stress status of calves (**C**).

Accordingly, the indirect heat stress effects on calves are presented in Fig. 3. For calves, 22,942 genes with nonzero total read count were examined. Among the genes with adjusted *p*-value (output from the DGE analysis) lower than 0.1, 472 genes were upregulated (LFC > 0), while 183 genes were downregulated (LFC < 0). In total, we detected 94 significantly differentially expressed genes (adjusted *p*-value < 0.05 and |LFC|> 0.58), of which 83 genes showed upregulation, while 11 genes were downregulated (Fig. 3A). The differences between two calve groups according to the indirect maternal HS status indicated no clear trend for the first 20 significant genes (Fig. 3B). A few samples displayed extremely strong increases in read counts for some genes (Fig. 3C). Similar to the direct heat stress analysis in dams, 48% of genes were excluded from the calf analyses due to low expression levels, i.e., a mean read count of < 13.

**Fig. 4:**
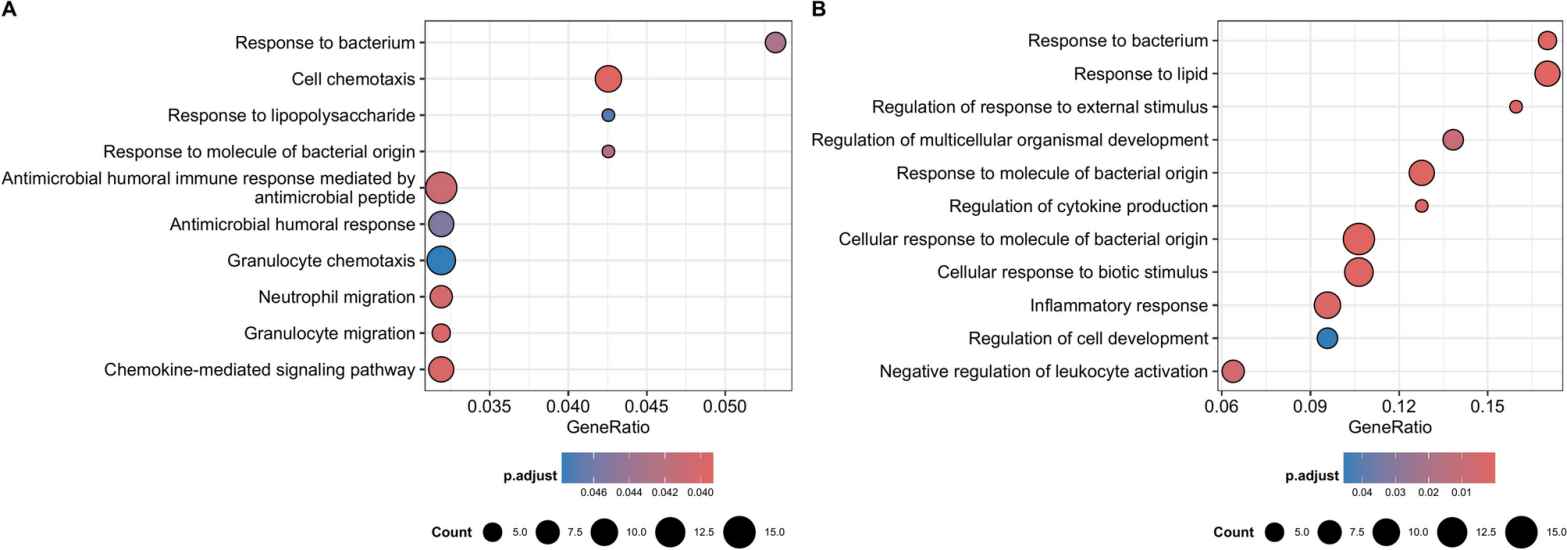
Overrepresented biological processes with respect to a false discovery rate < 0.05 in heat stressed dams (direct heat stress effects) and in indirectly heat stressed calves (indirect maternal heat stress effects) from GO enrichment analyses (**A**), and biological processes with regard to indirect maternal heat stress in calves detected via gene set enrichment analyses and GO enrichment analyses (**B**).

The ongoing GO enrichment analyses contributed to the detection of 10 significantly overrepresented biological processes in heat stressed dams, and of 95 overrepresented biological processes with regard to indirect maternal heat stress in calves (Table S2). Interestingly, all biological processes identified in dams were also significant in the time-lagged calf study. The respective 10 biological processes are illustrated in Fig. 4A. The GSEA application inferred 94 significant biological processes in calves (Table S2), and 11 of these processes were confirmed via GO enrichment analyses (Fig. 4B). No significant biological process were inferred with regard to direct HS in dams. Two biological processes, including the response to molecules of bacterial origin and response to bacteria, were significant in dams and in calves as well across the two methodological approaches as applied in the calf study. The genes *CXCL1*, *CXCL2*, *CXCL3*, *IL10*, *SNAI1*, *NR4A1* and *PTGS2* significantly contributed to biological processes with regard to direct heat stress in dams and indirect maternal heat stress in calves.

### Multi-omics analyses

**Fig. 5:**
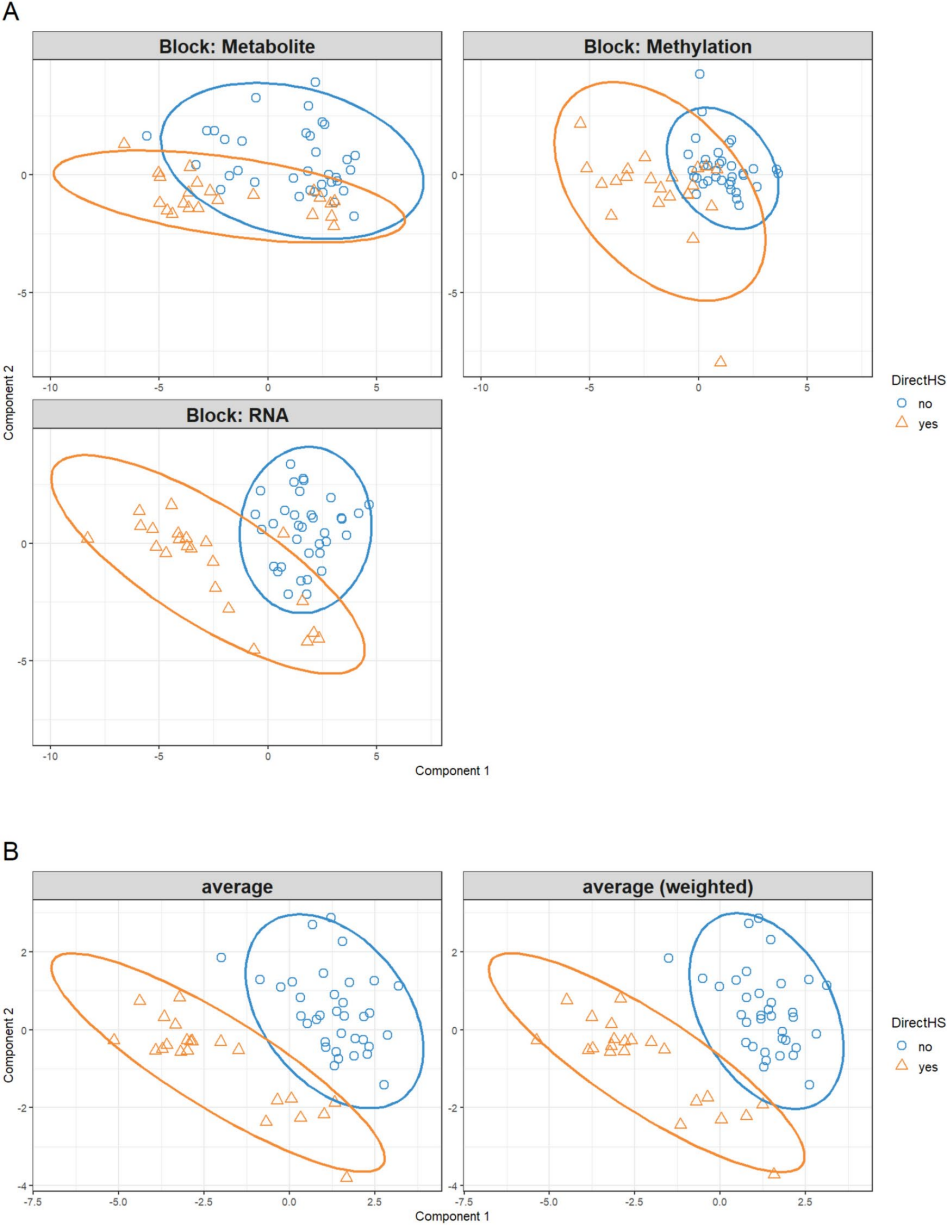
Plot of the first and second principal components for metabolite data, methylation data and gene expressions (RNAseq) (**A**), and for the average and weighted averages considering from three “omics” data (**B**).

With regard to direct HS in dams and the first PCs of the different “omics” features, the correlation coefficient was 0.45 between methylation and gene expression data, 0.62 between expression and metabolites, and 0.38 between methylation and metabolite data (Fig. S1). Based on the first components of the three datasets, we observed clear separations between the dams with direct HS and the respective control group, in particular when combing methylation with the other omics datasets (Fig. S1). With regard to the second components, the correlations slightly decreased, and both dam groups are not clearly separated (Fig. S1). With focus on the first and second components of the three multi-omics datasets simultaneously, it is possible to distinguish between dams with and without HS, especially with regard to the utilized gene expression data (RNAseq) in clustering approaches (Fig. 5A). The separation of the two groups was very obvious when using the average and weighted average of the first and second components from the three multi-omics datasets (Fig. 5B). The “average strategy” simply averaged the first and second components of the three blocks, while the “weighted average strategy” considered the correlations between the first two components and the direct HS status of the dams. Both strategies indicate a very similar discriminative ability based on our “omics”-datasets.

In total, 25 features from each multi-omics data were selected to generate PC 1, but for PC 2, methylation percentages in 6 promoters, gene expression levels of 15 genes and 7 metabolites, were selected. Contributions of the selected features to PC 1 and PC 2 are shown in Fig. S2A and B, respectively. The correlations of each selected feature to the first and second components is illustrated in Fig. S3. Among the selected features from each “omics block”, 5 promoter regions and 8 genes showed strong negative correlations with component 1 (correlation < − 0.5). One promoter, 19 genes and 22 metabolites strong positively correlated with component 1 (correlation > 0.5). For the second component, one promoter and 6 genes showed strong positive contributions, while 2 promoters, 5 genes and 5 metabolites were strongly negatively correlated with principal component 2. Hence, methylation percentages in 9 promoters, RNA expressions of 37 genes and 27 metabolites substantially contributed to the generation of first and second components in determining the dams with and without direct HS.

**Fig. 6:**
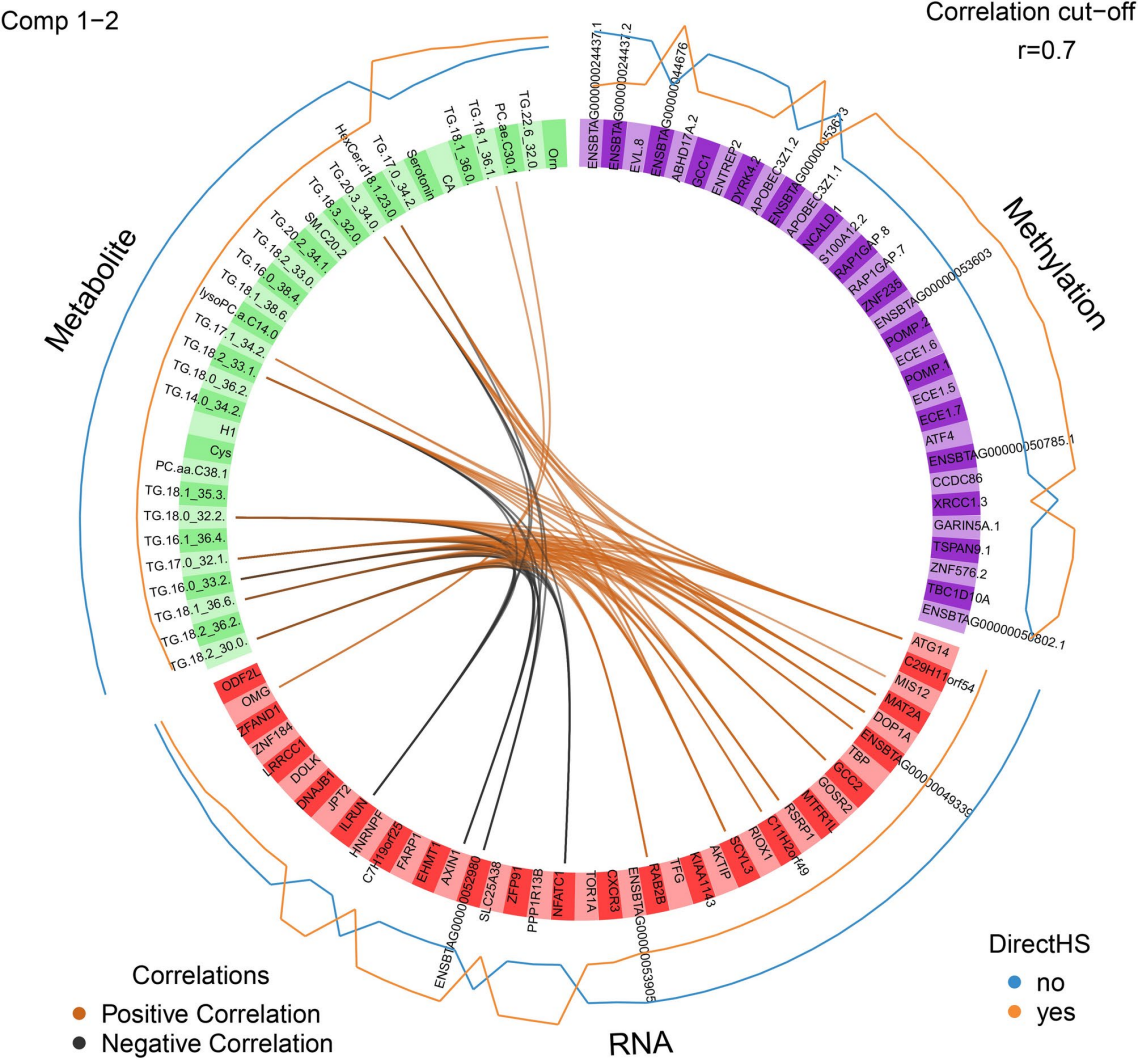
Correlation coefficients among the selected features of the three types of “omics data” (metabolite, RNAseq, methylation). Only associations for correlation coefficients >|0.7| are displayed.

**Fig. 7:**
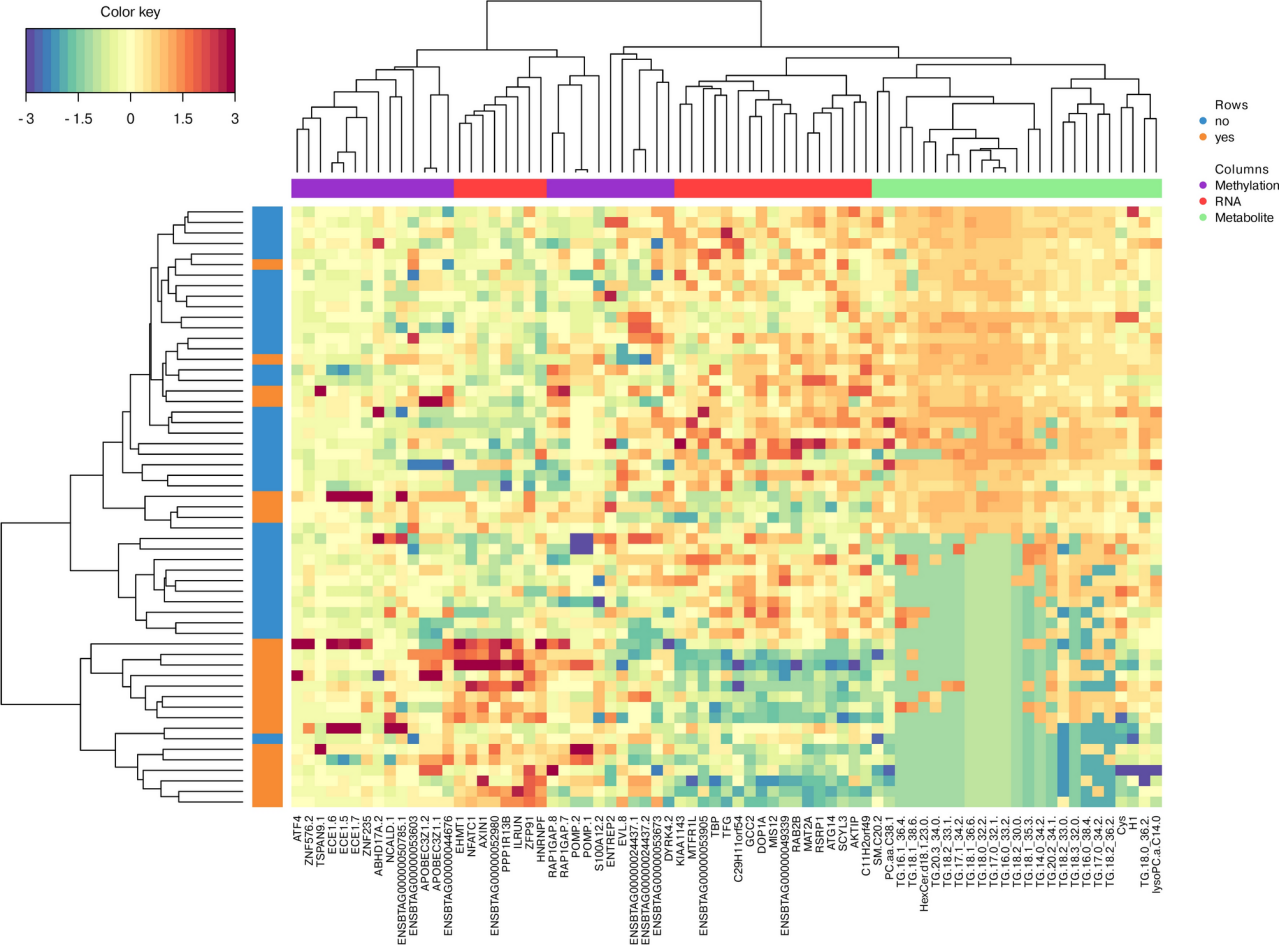
Heat map for the different “omics features” from methylations, RNAseq and metabolites contributing to principal component 1 in heat stressed dams (rows indicated with yes = in orange) versus the respective control group (rows indicated with now = in blue).

The correlation coefficients among the selected features of the three types of “omics data” are presented in Fig. 6. Among the 15 genes displaying strong correlations with metabolites (correlation coefficient >|0.7|), expressions from the genes *ILRUN*, *AXIN1*, E*NSBTAG00000052980* and *NFATC1* were negatively correlated with several metabolites, while the expressions from remaining genes and metabolite measurements were positively correlated. Interestingly, all genes with expressions being negatively correlated with metabolite measurements, displayed higher expression levels in direct HS dams compared to the respective control group. A complete overview of connections between genes and metabolites is listed in Table S3. Methylation was not closely correlated with any expression and metabolite measure. Figure 7 refers to the heat map of the different “omics features” for PC 1 selected by multiblock sPLS-DA (Fig. 7). The features mainly contributing to PC 1 could primarily classify the two dam groups (i.e., directly heat stressed dams versus control group). The dam group with direct heat stress clearly indicated low concentration of the 25 selected metabolites, along with 17 slightly downregulated and eight slightly upregulated genes. Methylation levels of the 25 promoter regions indicated “mixed pattern” in both dam groups (direct heat stress group and control group).

The performance of the first two components of the selected “omics features” was assessed using tenfold cross-validation with 10 replicates. In the dam control group without direct HS, the error rate was 10.57%. The corresponding error rate for direct HS dams was 33.64%, with an overall balanced error rate of 22.10%. The error rates decreased by additionally considering the second component. The areas under the curve were 0.67, 0.99, and 0.97 when using methylation, RNA, and metabolites, respectively. Hence, compared to the other omics datasets, methylation information is the least important omics tier to classify the dams in terms of direct HS. The balanced error rate in the test set with no methylation data of 28 dams was 24.44%, which was slightly higher than the balanced error rate from the tenfold cross validation.

## Discussion

We hypothesized differing metabolic profiles, DNA methylations and gene expressions differs between dam and calve groups, and also between directly or maternally heat stressed animal groups when compared with the respective control groups. From the intergenerational perspective, we found obvious clustering in the transcriptomic and metabolic profiles between dams and calves. In the context of HS separation, PCA enabled a clear separation of HS and control groups, especially when using the gene expression data. A quite large number of differentially expressed genes and differentially methylated promoters was identified in the dams with regard to direct HS effects, and in calves with regard to indirect HS effects. Such results indicate different genomic and physiological mechanisms at different ages and in different environmental climatic conditions, supporting our hypothesis.

### Principal component analyses: intergenerational aspects

With regard to the exploration of the PCs based on GRM, methylation gene expression and metabolic profiles, obvious clustering between dams and claves was observed in transcriptomic and metabolic profiles, but not in GRM and methylation profiles. Hence, overlaps between dams and calves in PCs for the GRM and methylation profiles reflect inheritance and especially the existing genetic relationships between dam and offspring (as known from pedigree based analyses)^17^. However, from the across-generation perspective, the clear separation due to the PCs from the metabolome and transcriptome indicates the differing genetic and physiological mechanisms in newborn calves and in cows. In this regard, PC1 which explained 42.07% and 30.51% of the total variance of transcriptomic and metabolomics data, respectively, clearly separated the dams from the calves without partial overlapping. A complete separation of metabolic profiles between calf age and heifer age was similarly found by Kenéz et al.^18^. From an across-breed perspective and considering a transcriptomic approach at same ages, Surlis et al.^19^ reported differentiated gene expressions of beef and dairy calves.

### Differentially methylated promoters, gene expressions and biological pathways

Gene expressions are expected to be regulated by epigenetic modifications, such as microRNAs, long non-coding RNAs, DNA methylation and histone modifications, with impact on phenotypic variation^20,21^. One of the most studied epigenetic modification controlling gene expression is DNA methylation, a modification which mostly occurs at the cytocine of a CpG dinucleotide to form a CG (or GpG) dinucleotide^22^. This modification goes along with the adsorption of a methyl group at the carbon 5 position of a cytosine nucleotide^23^. A CpG island, which is enriched for CpG dinucleotides, is frequently located in promoter regions at the 5’ of coding sequences^24^. DNA methylation can either up-regulate or suppress transcription, depending on its location in the genome. Hence, when located in regulatory regions (e.g. promoters) increased DNA methylation silences downstream genes^25^. In the context of long-term adaptation to climatic conditions, sheep and goats showed differential DNA methylation, when reared under two contrasted climates^26^. Similarly, altered DNA methylations have been associated with climatic stress response in cattle^24,27,28^. In this regard, Del Corvo et al.^24^ identified a total of 1967 differentially methylated regions (DMRs) when comparing blood samples of a climatically stressful (no shade) and a recovery period (shade) in Angus and Nellore bulls. However, only 214 of DMRs were observed in both breeds, indicating that heat stress related epigenetic signatures are mainly breed specific. Also, more than 50% of DMRs were located in introns and 31% were located in exons, with less than 5% in each of 3′, 5′ untranslated regions (UTR) and promoter regions. In the GO enrichment analysis, Del Corvo et al.^24^ found differentially methylated genes within the GO pathways *cellular process, single-organism process* and *metabolic process* in both breeds. In this regard, they identified several genes significantly involved in the biological processes of the immune system and inflammatory response. Sajjanar et al.^28^ identified distinct genome-wide methylation patterns and differentially expressed genes (DEGs), indicating epigenetic regulations inducing differences in thermotolerance between *Bos indicus* cattle and *Bos indicus* x *Bos taurus* crossbreds. In total, 79 genes showed both, i.e., differential methylation and differential expression. These genes were involved in cellular stress response. Livernois et al.^27^ examined genome-wide DNA methylation of blood mononuclear cells from high and low immune responder cows in response to HS. They found differentially methylated gene promoters in samples from heat stressed high immune responder cows associated with stress response and apoptosis prevention, and differentially methylated gene promoters in heat stressed low immune responder cows associated with cell proliferation and histone deacetylases.

Integrating the above mentioned theoretical framework and related genomics climate research in the context of the direct HS status of dams in our present study, we found 5 significantly hypermethylated promoters in upstream regions of the genes *LSAMP, ENSBTAG00000051923, GIMAP8, ENSBTAG0000005142 and EBD*. The gene *LSAMP* (limbic system associated membrane protein) is involved in emotional functions^29^ and has been upregulated in heat stressed male Duroc pigs^30^. The gene *GIMAP8* belongs to the *GTPase* family of immunity-associated proteins (GIMAPs). It is predominantly expressed in immune cells like T and B cells^31^ and has been identified as major candidate gene for tick resistance in cattle under tropical conditions^32^. The gene *EBD* (enteric β-defensin) belongs to the cluster D of the β-defensin gene family, with the ability to stimulate chemotaxis^33^. In broiler chickens, heat stress decreased avian β-defensins, innducing chicken susceptibility to invasion of different organs by *Salmonella Enteritidis*^34^. *ENSBTAG00000051923* and *ENSBTAG0000005142* are Ensembl gene identifiers with no names (unnamed genes). For clarification of the roles of proteins encoded by these genes, further studies are required. They might be involved in mechanisms reacting to heat stress in cattle. However, we did not find a significantly overrepresented biological process in the GO enrichment analysis. We found 19 significantly different expressed genes between the two direct HS groups of dams, with five of them downregulated. Nevertheless, the significant differences mainly based on two samples (HS.IU_1630 and HS.IU_1842), affecting the validity of the results.

With regard to indirect maternal HS, the “key study” by Skibiel et al.^9^ clearly indicated that HS during late gestation strongly contributed to epigenetic alterations with regard to DNA methylation. Indeed, Skibiel et al.^9^ identified more than 1,500 CpG sites differently methylated in liver of bull calves and mammary glands of heifers, which were either in utero heat stressed or cooled. The CpGs were associated with approximately 400 genes, which were identified to play a significant role in the pathways related to transcription, immune function, cell signaling, enzyme activity, cell cycle and *cell development* in bulls, and in the pathways *protein binding*, *phosphorylation, enzyme and cell activation* and *cell signaling* in heifers. Interestingly, there were 50 differentially methylated common genes that were shared by both groups, bull calf liver and heifer mammary gland, suggesting that indirect maternal HS may program different organs in a similar manner. In our study, we found the unnamed gene *ENSBTAG00000051923* being differentially methylated in both HS groups (direct HS dam group and indirect HS calf group), when compared to the respective control groups, indicating, that direct and indirect HS may affect specific genes, independent from age. Also other stress components seem to act similarly as HS. In Brahman bull calves, which were prenatally stressed by transportation, 113 pathways related to *behavior, stress response, neural function, metabolism, immune function, cell signaling* and other biological processes were significantly altered, when compared to controls (no prenatal transportation stress)^35^. In the direct HS status group of dams and in the indirect HS status group of calves, we did not find significantly overrepresented biological processes via GO enrichment analysis. However, there was one hypermethylated promotor due to indirect HS in calves, i.e., *AP1B1* (adaptor related protein complex 1 beta 1 subunit). *AP1B1* encodes lysosomal enzymes, and its expression was decreased in cattle during an infection with Johne’s disease (*Mycobacterium avium* subsp*. Paratuberculosis*^36^. With regard to gene expressions, we found 94 significantly differently expressed genes between the two indirect maternal HS groups of calves, with 11 of them downregulated.

In the ongoing GO enrichment analyses, we identified 10 significantly overrepresented biological processes in heat stressed dams and calves, reflecting similar pattern. The two biological processes “response to molecules of bacterial origin” and “response to bacteria” were also significant in the GSEA application for calves and dams. The seven genes *CXCL1*, *CXCL2*, *CXCL3*, *IL10*, *SNAI1*, *NR4A1* and *PTGS2* contributed significantly to the biological processes. In this regard, *CXCL1*, *CXCL2* and *CXCL3* belong to the Gro cluster of the CXC chemokine family, of which almost all are inflammatory chemokines^37^. *CXCL1* has been proposed as a biomarker and therapeutic target in mastitis^38^. Besides *CXCL1*, also *CXCL2* and *CXCL3* homologues have been increased in *Staphylococcus aureus* lipoteichoic acid-induced mastitis^39^, and expressions of *CXCL1* and *CXCL3* were increased following *Salmonella* infection in cattle^40^. *IL10*, an anti-inflammatory cytokine was upregulated in dairy cows during HS^41^. The *SNAI1* gene has been associated with black coat color in camels^42^. *NR4A1* (nuclear receptor subfamily 4, group A, member (1) was associated with cell death and was upregulated in heat stressed Karan-Fries cattle^43^. *PTGS2* (Prostaglandin-endoperoxide synthase (2) was identified as a hub gene, which is a possible biomarker of the immune response in dairy cattle under heat stress^44^.

### Multi-omics analyses

With regard to our multi-omics-analyses, we were able to distinguish between dams with and without HS when considering the first component of the three different “omics” features and when considering the average and weighted average of the first and second components from the three datasets. In total, methylation percentages in 9 promoters, RNA expressions of 37 genes and 27 metabolites substantially contributed to the generation of first and second components, determining the dams with and without direct HS. While methylation was not highly correlated with any expression and metabolite, we found strong negative correlations for the expressions of the four genes *ILRUN*, *AXIN1*, *ENSBTAG00000052980* and *NFATC1* with metabolite measurements, displaying higher expression levels in direct HS dams when compared to the respective control group. The gene *ILRUN* is known to act as an inhibitor of antiviral and proinflammatory cytokine transcription^45^. *AXIN1* expression, a transcript belonging to the growth and development group, was 1.2-fold increased in lactating dairy cows with intramammary infusion of lipopolysaccharide, what triggered an inflammatory response, when compared to a control group^46^. *NFATC1* belongs to the *NFAT* gene family, where mediated transcription is induced in epidermal cells in response to UV light^47^. *NFATC1* encodes the NFAC1 protein that plays a role in the inducible expression of cytokine genes in T cells^48^, and was identified as one of nine promising candidate genes for susceptibility to paratuberculosis in dairy cattle^49^.

The performance assessment of the first two components of selected omics features by tenfold-cross-validation with 10 replicates showed areas under the curve of 0.67 for methylation, 0.99 for RNA and 0.97 for metabolites. These results indicate methylation information as the least important omics tier to classify the dams in terms of direct HS, when compared to the other omics datasets.

The main results from the present study supported our scientific hypothesis. However, the study also had some limitations. In this regard, some effects could not be standardized in the study design (e.g. day of blood sampling), but the blood sampling date was considered as explanatory variables in the statistical model. As described, we focused on the direct effects of HS on the day before blood sampling or on the maternal HS during the last 8 weeks of gestation. Nevertheless, HS during earlier stages of gestation might have had an effect on metabolism, methylation and gene expression as well. Additionally, the chosen HS threshold of THI 60 based on previous comprehensive evaluations in lactating cows kept in different production systems^50^, but thresholds might differ in very late pregnancy stages or in calves.

## Conclusions

With regard to the intergenerational perspective, transcriptomic and metabolic profiles showed obvious clustering between dams and calves, indicating the different genomic and physiological mechanisms at different ages. With focus on the different “multi-omics” tiers, and in the context of HS separation, gene expression and methylation data clearly separated HS and control groups. In the multi-omics analysis, methylation information was the least important omics tier to classify the dams in terms of direct HS, when compared to the other omics datasets.Overall, the present HS research considering the intergenerational perspective indicated novel insights into the stress mechanisms of the “omics-tiers” metabolites, gene expressions and methylations. Our results contribute to a deeper understanding of the HS response of dairy cattle and their female progeny on the different molecular levels. Regarding future HS mitigation strategies, the identified particularities in metabolite profiles, gene expressions and methylation pattern in HS and control groups can be used as individual biomarkers to define the onset of HS in cattle, being independent from the general environmental THI classification.

## Materials and methods

### Experimental design and blood sampling scheme

All experimental procedures performed in this study are approved by the local authority for animal welfare affairs (Landesuntersuchungsamt Rheinland-Pfalz, Koblenz, Germany, and are in accordance with the German Animal Welfare Act (permit number: A19-20-002 EV). This study is reported in accordance with ARRIVE guidelines (https://arriveguidelines.org).

Blood sampling was conducted at the Educational and Research Centre for Animal Husbandry, Hofgut Neumühle, Münchweiler a. d. Alsenz, Germany, from February 2020 to October 2022. During this period, a total number of 88 German Holstein dairy cows (dams) and their 93 female calves were blood sampled for DNA and RNA extraction and for metabolic phenotyping, i.e., to generate the data at the different “omics levels” including genome sequencing, methylation pattern, gene expressions (RNA reads) and metabolic profiles. Dams were from parities 1 to 5 (classified into the three parity classes 1, 2 and ≥ 3 for the statistical analyses) and were kept in a freestall dairy shed providing identical feeding (total mixed ration (TMR) as ad libitum diet) and husbandry conditions. All dams were dried off exactly seven weeks before the expected calving date. All calves were kept in straw-bedded single calf hutches from birth until day 10 of life and received an identical colostrum and milk replacer program. To ensure a colostrum intake of 3 L from the respective dam within the first 2 h after birth, the calving sensor system Moocall (Moocall LTDD, Bluebell, Dublin, Ireland) was used. The feed supply included fresh water ad libitum and small amounts of hay. Blood samples which failed the standard protocol guidelines for metabolic phenotyping or RNA extraction (e.g. due to technical problems) were excluded from the ongoing analyses.

**Fig. 8:**
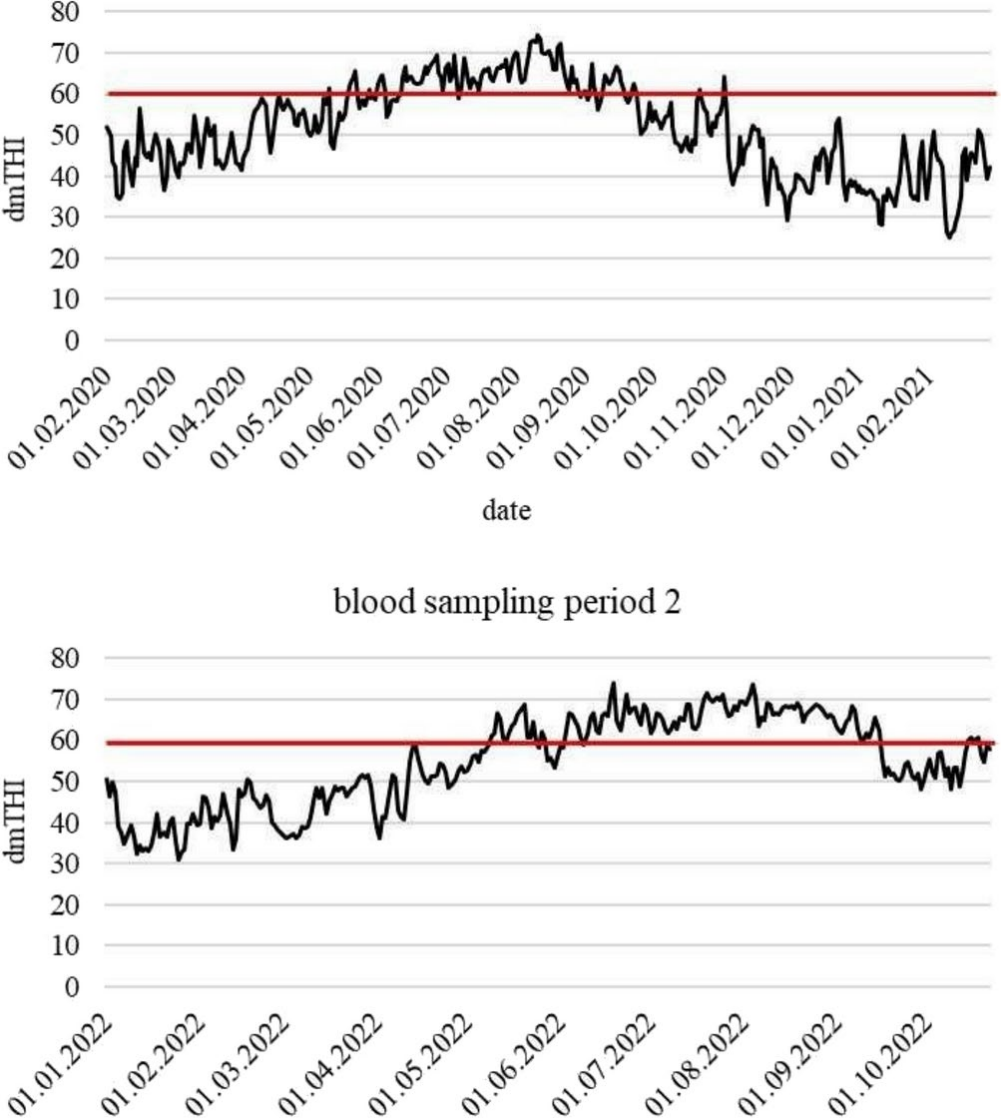
Overview of the daily mean THI values (dmTHI) recorded during blood sampling period 1 and 2 and heat stress threshold (dmTHI ≥ 60) (indicated as red line).

The general experimental design for blood sampling in dams and calves at different periods with respective animal numbers is illustrated in Fig. 8. In the framework of this design, dry dams were blood sampled during the last 12 d before calving. Their female calves were considered for blood sampling until the age of 2 weeks. Blood samples were collected from February 2020 to February 2021 (blood sampling period 1) and from January 2022 to October 2022 (blood sampling period 2). Sampling was conducted between 09:00am and 17:45 pm. In 2020 and early 2021, blood sampling comprised 60 dams (G0 dam generation) and 65 female calves (G1 calf generation). Two years later, 28 G1 females (G1 dam generation) were used for blood sampling for a second time during late gestation, and their 28 female calves (G2 calf generation) were blood sampled during the first 2 weeks of age. The final dataset included 88 dams (G0 dams + G1 dams) and 93 female calves (G1 calves + G2 calves). Blood samples were taken from the tail vein and collected between 0900 and 1745 h into 2 EDTA tubes (S-Monovette, Sarstedt AG & Co, Nürnberg, Germany). In a first step, a small amount of blood was transferred into 1.5 ml collection vials immediately after sample collection, and frozen at − 20 °C for later DNA extraction, performed in the laboratory of Justus-Liebig-University, Gießen, Germany. In a second step, a small amount of blood was prepared for later RNA extraction. In this regard, 1 ml of blood was suspended with 5 ml EL-buffer (lysis buffer) and incubated on ice for 10 to 15 min. Afterwards, the suspension was centrifuged (1128 × g, 10 min, 4 °C), forming a pellet of leukocytes. The supernatant was discarded without disturbing the pellet and the pellet was re-suspended with 2 ml EL-buffer and centrifuged again for 10 min, using the same settings as before. After discarding the supernatant a second time, the pellet was suspended with 600 µl of a RLT-buffer suspension (buffer for lysis of cells and tissues before RNA isolation, mixed with 2-Mercapthoethanol (2-ME) (10 µl 2-ME/1 ml RLT-buffer) and frozen at − 80 °C. Frozen samples were stored on dry ice and sent to the Gene Center of the Ludwig-Maximilians-University, Munich, Germany, where RNA extraction was performed. In a third step, the remaining collected blood was centrifuged (2500 × *g*, 10 min, 20–24 °C) to separate cells and plasma, following the guidelines for blood sample preparation for metabolic phenotyping (Biocrates Life Sciences AG, Innsbruck, Austria). Afterwards, blood plasma was transferred into pre-cooled storage vials and immediately frozen at − 80 °C. Frozen samples were also stored on dry ice and sent to Biocrates Life Sciences AG for metabolomics profiling.

### Climate data collection, HS classification and animal grouping

**Fig. 9:**
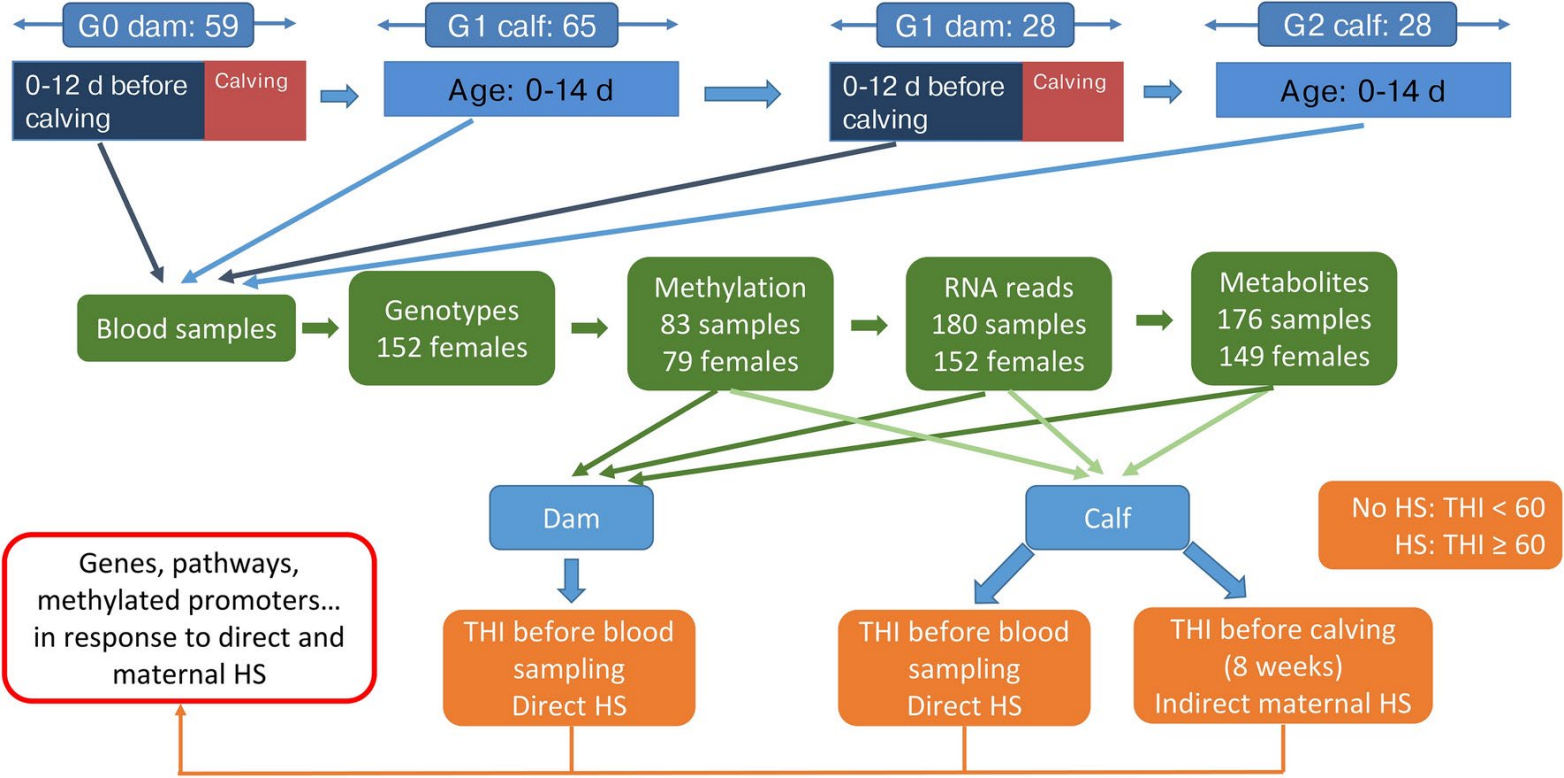
Overview of the general direct heat stress design in dams and indirect maternal heat stress design in calves considering different generations (G0, G1, G2) with respective animal and blood sample numbers.

Electronic data loggers (Tinytag Plus 2 TGP-4500, Gemini Data Loggers) were installed in the dairy shed to record climate data (temperature (°C) and humidity (%)) in 1 h intervals. Applying the formula of the National Research Council (NRC)^51^, the hourly temperature humidity index (THI) was calculated as follows:

$${\text{THI}} = \left[ {\left( {{1}.{8} \times {\text{T}}\left( {^\circ {\text{C}}} \right)} \right) + {32}} \right] - \left[ {0.{55}{-}\left( {0.00{55} \times {\text{RH}}\left( \% \right)} \right)} \right] \times \left[ {\left( {{1}.{8} \times {\text{T}}\left( {^\circ {\text{C}}} \right)} \right){-}{26}} \right],$$

where T is the dry bulb temperature and RH is the relative humidity. Thereafter, we used the hourly THI to calculate a daily mean THI (dmTHI) and a weekly mean THI (wmTHI). Direct HS was defined as dmTHI ≥ 60 before the blood sampling day for dams and calves. The definition of the HS-threshold dmTHI ≥ 60 based on the results by Brügemann et al.^50^ who defined and evaluated HS thresholds for the same THI formula in lactating Holstein dairy cows under German climatic conditions. The dmTHI values recorded through the study are shown in Fig. 8. Indirect maternal HS refers to the time lagged HS effects during the last eight weeks of gestation on G1 and G2 calves. In case of the averaged wmTHI ≥ 60, the calf underwent HS, otherwise not. The allocation of THI to blood sampling dates is outlined in Fig. 9. Table 1 shows the complete classification of HS statuses based on THI values and day/period of classification for dams and calves.

**Table 1:** Classification of heat stress (HS) status based on daily mean THI (dmTHI) and average weekly mean THI (wmTHI) values and day/period of classification for dams and calves.

		Dams	Calves
Direct HS	HS threshold	dmTHI ≥ 60	dmTHI ≥ 60
	HS classification day	Day before blood sampling	Day before blood sampling
	HS classification	dmTHI ≥ 60 = HS	dmTHI ≥ 60 = HS
		dmTHI < 60 = no HS	dmTHI < 60 = no HS
Maternal HS	HS threshold		Average wmTHI ≥ 60
	HS classification day		Last 8 weeks of gestation
	HS classification		Average wmTHI ≥ 60 = HS
			Average wmTHI < 60 = no HS

### Laboratory analyses

#### DNA and RNA extraction

Genomic DNA was extracted using the Macherey–Nagel™ NucleoSpin™ Blood Kit at the laboratory of the Justus-Liebig-University, Gießen, Germany. According to the manufacturer’s protocol, 2 ml of blood was used. DNA with an absorbance ratio at 260 and 280 nm of ~ 1.8, representing pure DNA, was standardized to 80 to 120 ng/µl using the Nanodrop 1000 spectrophotometer (Peqlab, Erlangen, Germany). For further analysis steps (e.g. nanopore sequencing), DNA was sent to the Gene Center of the Ludwig-Maximilians-University, Munich, Germany, where also RNA isolation was performed.

For RNA isolation, 100 µl of the cell suspension, which was prepared in step two of blood sample preparation, was blended with a RLT-buffer—TriZOL (900 µl) mixture and homogenized with a Heidolph SilentCrusher. Afterwards, the suspension was purified and eluted using the Direct-zol RNA Miniprep (Zymo Research Europe GmbH, Germany).

### RNA sequencing

Whole blood was treated with RBC lysis buffer and conserved in RLT. Total RNA was isolated using the Zymo directZol Mini Prep kit by homogenizing 100 ul of RLT cell suspension in 900 ul TriZOL reagent supplemented with 20 ul 1-Thioglycerol. The RNA concentration was determined using a Nanodrop spectrometer (Thermo Fisher Scientific, Waltham, MA, USA), and RNA quality was assessed using an Agilent Bioanalyzer (Agilent RNA 6000 Nano kit, Agilent Technologies, Santa Clara, CA, USA).

Libraries were prepared using the Lexogen SENSE mRNA-seq Library Prep Kit V2 (Lexogen, Greenland, NH, USA). In summary, 200 ng of total RNA were captured on oligo-dT beads and reverse transcribed directly on the beads, followed by the synthesis of the second cDNA strand after adapter ligation. The adapted cDNA was eluted and then amplified with barcoded primers through 12 cycles of PCR, using the following conditions: 98 °C for 30 s, 65 °C for 20 s, 72 °C for 30 s, and a final extension at 72 °C for 1 min (hold at 10 °C). PCR products were purified using Agencourt AMPure XP beads (Beckman Coulter, Brea, CA, USA). The quality of the libraries was verified with an Agilent Bioanalyzer (DNA 1000 Kit). The finalized barcoded libraries were pooled and sequenced on an Illumina NextSeq 2000 (Illumina, San Diego, CA, USA) on P3 flowcells with a read length of 75 nucleotides in single-end mode.

### DNA-methylation

High molecular weight dsDNA was isolated from whole blood using the Macherey&Nagel kit XX. 200 ng of DNA were further purified with AmpureXp beads, eluted in 12,5 ul nuclease-free water and treated with NEBnext Ultra II end-repair and A-tailing module (E754, New England Biolabs, Ipswich MA, USA), ligated to barcode adapters (Native Barcoding kit 24, Oxford Nanopore Technologies, Oxford, UK), purified and measured by qubit. Equal amounts of each barcoded library were pooled in groups of 16 and ligated to native sequencing adapter (Native barcoding kit v14, Oxford Nanopore Technologies, Oxford, UK). 150 ng of final library were loaded on PromethION 114 M flowcells (R10.4.1 version) and sequenced for 72 h using the adaptive sampling method providing a BED file. This BED file contained the genomic loci of the genes previously identified as candidate regions, padded with 25 kb on each side, plus all the annotated CpG islands (also padded with 25 kb) in a radius of 200 kb around the candidate regions as potentially regulatory sites. Each sequenced read was immediately basecalled, mapped and if the mapping coordinates matched the BED file, sequencing was continued until the end of the DNA fragment. All non-matching reads were immediately rejected by voltage reversal, corresponding to a read length of 500–600 bp.

Nanopore sequence data were used for the ongoing single-nucleotide variants (SNVs) and for CpG methylation analyses.

### Metabolomic profiling

Metabolomic profiling was carried out using a targeted quantitative metabolomics approach employing the MxP Quant 500 Kit (Biocrates Life Sciences AG), identifying and quantifying 630 different metabolites of 26 biochemical classes. A detailed list of metabolites is provided in the supplemental material (Table S4). Concentrations (µM/L) of all metabolites were determined via mass spectrometry, as described in Halli et al.^2,52^.

### Calling variants in RNA-seq data

To explore the genomic relationships among animals, we identified short variants (SV; SNVs and Indels) in the RNA-seq data. First, we combined two fastq files from the same animal into one file, implying a decrease of RNA-seq samples from 180 to 152. Because blood samples for 28 female cattle were collected twice, i.e., after birth and before first calving. Afterwards, we clipped adaptors for RNA reads and discarded short reads (< 36 bp) and reads with poor quality according to the criteria implemented in cutadapt V4.2^53^. Afterwards, using the clean reads, 31,925 biallelic SV on autosomes were identified according to the workflows for processing RNA data with GATK V4^54^. Using this workflow and in case of 2 indel within a 25 bp segment, we discarded the indel with the lower overall quality (QUAL) score. The same strategy was applied to SNP, i.e., excluding the SNP with lower QUAL score within a segment of < 3 bp. Finally, 30,952 SV after filtering were used as a data basis for constructing a genomic relationship matrix (GRM) among the 152 female cattle according to the algorithm by Yang et al.^55^. Aftwerards, only first three principal components (PC) of the GRM calculated via GCTA^56^ were considered in the subsequent differential and association analyses to adjust for possible population stratification.

### DNA methylation analyses

The methylation levels of cytosines of 75 samples were stored in bedMethyl files separately. We only used sites with a minimum read coverage of 5 to perform the ongoing methylation analyses by applying the R package “methylKit”^57^. The transcription start sites for 41,843 transcripts available in the Ensembl database were identified, and a segment which comprised 2000 bases upstream plus 200 bases downstream of a transcription start site was defined as the promoter region of the transcript using GenomicFeatures^58^. Methylation sites located within the promoter regions were retained to count the number of detected bases per promoter region. We only computed methylation levels for 7364 promoter regions with a minimum of five detected bases. Principal component analysis (PCA) on the methylation profiles of the promoters was performed and the corresponding structure of the methylation data is illustrated in Fig. 1B.

Among the 75 blood samples, 57 samples were collected before calving from dams and the remaining 18 samples were from newborn calves. The 57 dams were classified into 2 groups with regard to the daily THI before blood sampling, i.e., 22 dams were assigned to the direct HS group and 35 dams to the control group (no direct HS). We removed promoter regions which only were identified in less than 10 cows per dam group (i.e., HS group and control group). Consequently, 841 regions were retained to identify differentially methylated promoters between dams with and without direct HS. In addition to the direct HS status, further confounding variables were considered to adjust for effects that might influence the methylation levels. In this regard, a generalized linear model with a logit link function as implemented in the calculateDiffMeth function of the “methylKit” package was applied to determine the impact of direct HS on methylation levels in promoter regions. The statistical model 1 to infer the direct HS effects in dams was defined as follows:

1 $$\begin{aligned} {\text{logit}}\left( {\uppi _{{{\text{ijkl}}}} } \right) = & {\text{log }}\left[ {\uppi _{{{\text{ijkl}}}} /\left( {1 - \uppi _{{{\text{ijkl}}}} } \right)} \right] = \upvarphi + {\text{CY}}_{{\text{i}}} + {\text{CS}}_{{\text{j}}} + {\text{LC}}_{{\text{k}}} + {\text{DAYS}} \\ & \quad + {\text{PC1}} + {\text{PC2}} + {\text{PC3}} + {\text{directHS}} - {\text{STATs}}_{{\text{l}}} \\ \end{aligned}$$

where π_ijkl_ = methylation percentages at a given promoter for 57 dams; φ = overall mean effect; CY_i_ = fixed effect of calving year (2020, 2021, 2022); CS_j_ = fixed effect of calving season (spring, summer, autumn, winter); LC_k_ = fixed effect of lactation class (1, 2, ≥ 3); DAYS = days between blood sampling date and calving date (0–12 days) as linear regression; PC1 to PC3 = first three PC of the GRM; directHS-STATs_l_ = fixed effect of direct HS status (0 = no direct HS = control group, 1 = direct HS). The model was repeated 841 times to estimate the effect of direct HS on each promoter.

The same analyses were performed considering 18 calves with methylation profiles. We also excluded promoters identified in less than 5 calves per indirect maternal HS group, resulting in 832 promoter regions. A value of 1 was assigned to calves with indirect maternal HS; otherwise, a calf received the score = 0 (control group without indirect maternal HS). The statistical model 2 to infer the indirect maternal HS effects in calves was defined as follows:

2 $$\begin{aligned} {\text{logit}}\left( {\uppi _{{{\text{ijkl}}}} } \right) = & {\text{log}}\left[ {\uppi _{{{\text{ijkl}}}} /\left( {1 - \uppi _{{{\text{ijkl}}}} } \right)} \right] = \upvarphi + {\text{BY}}_{{\text{i}}} + {\text{BS}}_{{\text{j}}} + {\text{directHS}} - {\text{STAT}}_{{\text{k}}} + {\text{AGE}} \\ & \quad + {\text{PC1}} + {\text{PC2}} + {\text{PC3}} + {\text{indirectMHS}} - {\text{STAT}}_{{\text{l}}} \\ \end{aligned}$$

where π_ijkl_ = methylation percentages at a given promoter for 18 calves; φ = overall mean effect; BY_i_ = fixed effect of birth year (2020 and 2022); BS_j_ = fixed effect of birth season (spring, summer); directHS-STAT_k_ = fixed effect of direct HS of the calf before blood sampling (0 = no direct HS and 1 = with direct HS); AGE = age of calves at blood sampling (0–14 days) as linear regressions; PC1 to PC3 = first three PC of the GRM, indirectMHS-STAT_l_ = fixed effect of indirect maternal HS status (0 = no indirect maternal HS = control group, 1 = indirect maternal HS). For all methylation analyses, promoters that exceeded a threshold of a *p*-value < 0.05 along with absolute differences of methylation percentages between test and control groups > 5%, were considered as significantly differentially methylated promoters.

### Differential gene expression analyses

The RNA-seq data was available for 180 samples, including 93 samples for calves and 87 samples for dams. Similar to the procedure we applied for variant calling, first we clipped adaptors for RNA reads and discarded short reads (< 36 bp) and reads with poor quality (< 20) applying the software package cutadapt V4.2^53^. In the second step, we assessed the quality of the RNA reads for each sample using FastQC V0.12.1^59^, and found that all samples past the per base sequence quality. Afterwards, the reference genome sequences and the annotation file according to bovine assembly ARS-UCD1.2 were provided to generate the genome index files using the software package STAR V2.7.11a^60^. Finally, the RNA-seq reads were mapped to the reference genome based on the index and genome files generated in the previous step and the number of reads per gene were counted for each sample using the flag “–quantMode GeneCounts” with STAR. After combining the reads per gene for all 180 samples, we identified 23,010 genes with nonzero RNA-seq reads. PCA was performed again based on 14,355 genes with variations in the read count, i.e., median absolute deviation (MAD) > 2.

In total, RNA reads from 85 dam samples were used in the differential gene expression (DGE) analyses using DESeq2 v1.40.2^61^. We removed the sample HS-IU.1804, because this sample was classified into the calf cluster (Fig. 1C). A generalized linear model for a negative binomial distribution with a logarithmic link function as implemented in in DESeq2 was used for DGE analyses. Fixed effects (in analogy with model 1) included in the model were calving-year-season (CYS, from 2020 to 2022 in combination with 4 seasons), LC, DAYS, PC1, PC2, PC3 and the directHS-STAT. The log2 fold changes (LFC) of the dams with direct HS relative to respective control group were shrunked using the apeglm function10 DESeq2 to visualize and rank the tested genes.

Similarly, RNA reads of 93 calves were analyzed with the same procedures using the DESeq2 package. The fixed effects (in analogy with model 2) included BY, BS, AGE, PC1, PC2, and PC3, directHS-STAT and indirectMHS-STAT.

For both analyses, i.e., in the dam as well as in the calf groups, significantly differently expressed genes were defined in case of an adjusted *p*-value < 0.05 for a false discovery rate < 0.05 and |LFC|> 0.58 (i.e., indicating a 1.5-fold increases or decreases in expressions) according to hbctraining^62^. The significantly differential expressed genes were further considered in GO enrichment analyses^63^ to detect excessively overrepresented biological processes with respect to a false discovery rate < 0.05. Additionally, all genes with valid p-values and LFC were ranked in terms of their LFC for dams and calves, respectively. The ranked genes were used in gene set enrichment analyses (GSEA) to identify GO biological processes that were significantly enriched compared to randomly ranked genes. The GSEA were carried out applying the gseGO function implemented in the R package “clusterProfile”^64^. Only the biological processes with a minimum of 10 genes and a maximum of 500 genes were plotted using the dotplot function from the R package “enrichplot”^65^.

### Multi-omics analyses

The methylation percentages of 841 promoter regions from 57 dams were further filtered in terms of *p*-value (< 0.05) and differences in methylation percentages between directHS-STAT groups (> 1%), resulting in the retention of 151 promoters for the multi-omics analyses. The normalized read counts from 85 dams generated by DESeq2 v1.40.2^61^ were included in the multi-omics analyses. In this regard, we used read counts for 2752 genes with *p*-value < 0.1. Regarding the metabolic profiles, we kept 288 metabolites which displayed a certain degree of variations across the 85 dam samples (specifically, SD > 0.1). 57 dams with three tiers of multi-omics data, including methylation, gene expression and metabolic profiles, were allocated to a training set. The remaining 28 dams with only available read counts and metabolite data, were allocated to a testing set. The multi-omics analyses integrated three tiers of multi-omics data by applying an integrated approach as implemented in DIABLO^66^ from the R package mixOmics v6.24.0^67^. In this regard, we explored features that maximize the separation of dams with and without directHS in the training set. The performance of the selected features was validated based on the prediction accuracy of the 28 dams in the testing set. Specifically, a tenfold cross validation repeated 10 times for up to five components were performed in the training set to identify the optimum number of components for the three types of multi-omics data. Based on balanced error rates of centroids distances in the classifications, the first two components of each multi-omics tier were sufficient for predicting the directHS-STAT of the dams in training set. In the next step, the optimal number of variables, including promoter regions, genes and metabolites, were selected in each multi-omics dataset. These variables were tuned, and then we used 25 features of the first component in all datasets. For the second component, numbers of features were set to 6, 15 and 7 for the methylation, gene expression and metabolomics data, respectively. Finally, only the first two components based on the aforementioned features, were used in the multi-omics analyses. Furthermore, we calculated Pearson correlation coefficients between PCs for PC1 and PC2 of the different omics-tiers.

## Supplementary Information

Supplementary Information 1.

Supplementary Information 2.

Supplementary Information 3.

Supplementary Information 4.

Supplementary Information 5.

Supplementary Information 6.

Supplementary Information 7.

Supplementary Information 8.

## Data Availability

The datasets generated and analyzed during the current study are available in the Gene Expression Omnibus (GEO) repository, accession number is GSE289946 at https://www.ncbi.nlm.nih.gov/geo/query/acc.cgi?acc=GSE289946. The token number for reviewer purpose is wnofqmyqfnezfev. The contact person in this regard for reviewer purpose is Dr. Tong Yin, email adr.: lizyincast@gmail.com

## References

[1] Mader, T. L., Davis, M. S. & Brown-Brandl, T. Environmental factors influencing heat stress in feedlot cattle. *J. Anim. Sci.* **84**, 712–719. 10.2527/2006.843712x (2006). 16478964 10.2527/2006.843712x

[2] Halli, K., Cohrs, I., Brügemann, K., Koch, C. & König, S. Effects of temperature-humidity index on blood metabolites of German dairy cows and their female calves. *J. Dairy Sci.* **106**, 7281–7294. 10.3168/jds.2022-22890 (2023). 37500442 10.3168/jds.2022-22890

[3] Kipp, C. et al. Across-generation effects of maternal heat stress during late gestation on production, female fertility and longevity traits in dairy cows. *J. Dairy Res.* **88**, 147–153. 10.1017/S0022029921000327 (2021). 33926583 10.1017/S0022029921000327

[4] Laporta, J. et al. Late-gestation heat stress impairs daughter and granddaughter lifetime performance. *J. Dairy Sci.* **103**, 7555–7568. 10.3168/jds.2020-18154 (2020). 32534930 10.3168/jds.2020-18154

[5] Jaenisch, R. & Bird, A. Epigenetic regulation of gene expression: How the genome integrates intrinsig and environmental signals. *Nat. Genet.* **33**, 245–254. 10.1038/ng1089 (2003). 12610534 10.1038/ng1089

[6] Strahl, B. D. & Allis, C. D. The language of covalent histone modifications. *Nature* **403**, 41–45. 10.1038/47412 (2000). 10638745 10.1038/47412

[7] Bartel, D. P. MicroRNAs: Target recognition and regulatory functions. *Cell* **136**, 215–233. 10.1016/j.cell.2009.01.002 (2009). 19167326 10.1016/j.cell.2009.01.002 PMC3794896

[8] Singh, K. et al. Epigenetics: A possible role in acute and transgenerational regulation of dairy cow milk production. *Animal* **6**, 375–381. 10.1017/S1751731111002564 (2011). 10.1017/S1751731111002564 22436216

[9] Skibiel, A. L. et al. In utero heat stress alters the offspring epigenome. *Sci. Rep.* **8**, 14609. 10.1038/s41598-018-32975-1 (2018). 30279561 10.1038/s41598-018-32975-1 PMC6168509

[10] Tao, S. E. E. et al. Short communication: Effect of heat stress during the dry period on gene expression in mammary tissue and peripheral blood mononuclear cells. *J. Dairy Sci.* **96**, 378–383. 10.3168/jds.2012-5811 (2013). 23141830 10.3168/jds.2012-5811

[11] Corazzin, M. et al. Effect of heat stress on dairy cow performance and on expression of protein metabolism genes in mammary cells. *Animals* **10**, 2124. 10.3390/ani10112124 (2020). 33207608 10.3390/ani10112124 PMC7696625

[12] Liu, G. et al. Effects of chronic heat stress on mRNA and miRNA expression in dairy cows. *Gene* **742**, 144550. 10.1016/j.gene.2020.144550 (2020). 32165293 10.1016/j.gene.2020.144550

[13] do Amaral, B. C. et al. Heat-stress abatement during the dry period: Does cooling improve transition into lactation?. *J. Dairy Sci.* **92**, 5988–5999. 10.3168/jds.2009-2343 (2009). 19923602 10.3168/jds.2009-2343

[14] do Amaral, B. C. et al. Heat stress abatement during the dry period influences metabolic gene expression and improves immune status in the transition period of dairy cows. *J. Dairy Sci.* **94**, 86–96. 10.3168/jds.2009-3004 (2011). 21183020 10.3168/jds.2009-3004

[15] Hasin, Y., Seldin, M. & Lusis, A. Multi-omics approaches to disease. *Genome Biol.* **18**, 83. 10.1186/s13059-017-1215-1 (2017). 28476144 10.1186/s13059-017-1215-1 PMC5418815

[16] Bersanelli, M. et al. Methods for the integration of multi-omics data: Mathematical aspects. *BMC Bioinf.* **17**(Suppl 2), 15. 10.1186/s12859-015-0857-9 (2016). 10.1186/s12859-015-0857-9 PMC4959355 26821531

[17] Moore, K. L., Vilela, C., Kaseja, K., Mrode, R. & Coffey, M. Forensic use of the genomic relationship matrix to validate and discover livestock pedigrees. *J. Anim. Sci.* **97**, 35–42. 10.1093/jas/sky407 (2019). 30329120 10.1093/jas/sky407 PMC6313117

[18] Kenéz, Á. et al. Different milk feeding intensities during the first 4 weeks of rearing dairy calves: Part 3: Plasma metabolomics analysis reveals long-term metabolic imprinting in Holstein heifers. *J. Dairy Sci.* **101**, 8446–8460. 10.3168/jds.2018-14559 (2018). 29935828 10.3168/jds.2018-14559

[19] Surlis, C. et al. Blood immune transcriptome analysis of artificially fed dairy calves and naturally suckled beef calves from birth to 7 days of age. *Sci. Rep.* **8**, 15461. 10.1038/s41598-018-33627-0 (2018). 30337646 10.1038/s41598-018-33627-0 PMC6194081

[20] Gibney, E. R. & Nolan, C. M. Epigenetics and gene expression. *Heredity* **105**, 4–13. 10.1038/hdy.2010.54 (2010). 20461105 10.1038/hdy.2010.54

[21] Allis, C. D. & Jenuwein, T. The molecular hallmarks of epigenetic control. *Nat. Rev. Genet.* **17**, 487–500. 10.1038/nrg.2016.59 (2016). 27346641 10.1038/nrg.2016.59

[22] Greenberg, M. V. C. & Bourc’his, D. The diverse roles of DNA methylation in mammalian development and disease. *Nat. Rev. Mol. Cell. Bio.* **20**, 590–607. 10.1038/s41580-019-0159-6 (2019). 31399642 10.1038/s41580-019-0159-6

[23] Wyatt, G. R. Occurrence of 5-methyl-cytosine in nucleic acids. *Nature* **166**, 237–238. 10.1038/166237b0 (1950). 15439258 10.1038/166237b0

[24] Del Corvo, M. et al. Methylome patterns of cattle adaptation to heat stress. *Front. Genet.* **12**, 633132. 10.3389/fgene.2021.633132 (2021). 34122501 10.3389/fgene.2021.633132 PMC8194315

[25] Tate, P. H. & Bird, A. P. Effect of DNA methylation on DNA-binding proteins and gene expression. *Curr. Opin. Genet. Def.* **3**, 226–231. 10.1016/0959-437X(93)90027-M (1993). 10.1016/0959-437x(93)90027-m 8504247

[26] Denoyelle, L. et al. Genetic variations and differential DNA methylation to face contrasted climates in small ruminants: An analysis on traditionally-managed sheep and goats. *Front. Genet.* **12**, 745284. 10.3389/fgene.2021.745284 (2021). 34650601 10.3389/fgene.2021.745284 PMC8508783

[27] Livernois, A. M., Mallard, B. A., Cartwright, S. L. & Cánovas, A. Heat stress and immune response phenotype affect DNA methylation in blood mononuclear cells from Holstein dairy cows. *Sci. Rep.* **11**, 11371. 10.1038/s41598-021-89951-5 (2021). 34059695 10.1038/s41598-021-89951-5 PMC8166884

[28] Sajjanar, B. et al. Genome-wide DNA methylation profiles regulate distinct heat stress response in zebu (*Bis indicus*) and crossbred (*Bos indicus* x *Bos taurus*) cattle. *Cell Stress Chaperon.* **29**, 603–614. 10.1016/j.cstres.2024.06.005 (2024). 10.1016/j.cstres.2024.06.005 PMC11264184 38936463

[29] Heimer, L. & Van Hoesen, G. W. The limbic lobe and its output channels: Implications for emotional functions and adaptive behavior. *Neurosci. Biobehav. R.* **30**, 126–147. 10.1016/j.neubiorev.2005.06.006 (2006). 10.1016/j.neubiorev.2005.06.006 16183121

[30] Srikanth, K. et al. Genome-wide transcriptome and metabolome analysis provide novel insights and suggest a sex-specific response to heat stress in pigs. *Genes* **11**, 540. 10.3390/genes11050540 (2020). 32403423 10.3390/genes11050540 PMC7291089

[31] Ciucci, T. & Bosselut, R. Gimap and T cells: A matter of life and death. *Eur. J. Immunol.* **44**(2), 348–351. 10.1002/eji.201344375 (2014). 24510500 10.1002/eji.201344375 PMC4005041

[32] dos Santos, C. G. et al. Candidate genes for tick resistance in cattle: A systematic review combining post-GWAS analyses with sequencing data. *J. Appl. Anim. Res.* **50**(1), 460–470. 10.1080/09712119.2022.2096035 (2022).

[33] Mackenzie-Dyck, S. et al. The synthetic peptides bovine enteric beta-defensin (EBD), bovine neutrophil beta-defensin (BNBD) 9 and BNBD 3 are chemotactic for immature bovine dendritic cells. *Vet. Immunol. Immunopathol.* **143**, 87–107. 10.1016/j.vetimm.2011.06.028 (2011). 21764462 10.1016/j.vetimm.2011.06.028

[34] Quinteiro-Filho, W. M. et al. Heat stress decreases expression of the cytokines, avian β-defensins 4 and 6 and Toll-like receptor 2 in broiler chickens infected with *Salmonella* Enteritidis. *Vet. Immunol. Immunop.* **186**, 19–28. 10.1016/j.vetimm.2017.02.006 (2017). 10.1016/j.vetimm.2017.02.006 28413046

[35] Littlejohn, B. P. et al. Prenatal transportation stress alters genome-wide methylation in suckling Brahman bull calves. *J. Anim. Sci.* **96**, 5075–5099. 10.1093/jas/sky350 (2018). 30165450 10.1093/jas/sky350 PMC6276578

[36] Heidari, M., Pakdel, A., Bakhtiarizadeh, M. R. & Dehghanian, F. Integrated analysis of lncRNAs, mRNAs, and TFs to identifiy regulatory networks underlying MAP infection in cattle. *Front. Genet.* **12**, 668448. 10.3389/fgene.2021.668448 (2021). 34290737 10.3389/fgene.2021.668448 PMC8287970

[37] Widdison, S. & Coffey, T. J. Cattle and chemokines: Evidence for species-specific evolution of the bovine chemokine system. *Anim. Genet.* **42**, 341–353. 10.1111/j.1365-2052.2011.02200.x (2011). 21749416 10.1111/j.1365-2052.2011.02200.x

[38] Sharifi, S. et al. Integration of machine learning and meta-analysis identifies the transcriptomic bio-signature of mastitis disease in cattle. *PLoS One* **13**(2), e0191227. 10.1371/journal.pone.0191227 (2018). 29470489 10.1371/journal.pone.0191227 PMC5823400

[39] Rainard, P., Fromageau, A., Cunha, P. & Gilbert, F. B. Staphylococcus aureus lipoteichoic acid triggers inflammation in the lactating bovine mammary gland. *Vet. Res.* **39**, 52. 10.1051/vetres:2008034 (2008). 18593548 10.1051/vetres:2008034

[40] Santos, R. L., Zhang, S., Tsolis, R. M., Bäumler, A. J. & Adams, L. G. Morphologic and molecular characterization of *Salmonella typhimurium* infection in neonatal calves. *Vet. Pathol.* **39**(2), 200–215. 10.1354/vp.39-2-200 (2002). 12009058 10.1354/vp.39-2-200

[41] Park, D. S. et al. Dynamic changes in blood immune cell composition and function in Holstein and Jersey steers in response to heat stress. *Cell Stress Chaperon.* **26**(4), 705–720. 10.1007/s12192-021-01216-2 (2021). 10.1007/s12192-021-01216-2 PMC8275816 34080136

[42] Bitaraf Sani, M. et al. Identification of candidate genes for pigmentation in camels using genotyping-by-sequencing. *Animals* **12**, 1095. 10.3390/ani12091095 (2022). 35565522 10.3390/ani12091095 PMC9104199

[43] Singh, A. K. et al. Genomewide expression analysis of the heat stress response in dermal fibroblasts of Tharparkar (zebu) and Karan-Fries (zebu x taurine) cattle. *Cell Stress Chaperon.* **25**, 327–344. 10.1007/s12192-020-01076-2 (2020). 10.1007/s12192-020-01076-2 PMC7058763 32062819

[44] Wang, J. et al. Involvement of the VEGF signaling pathway in immunosuppression and hypoxia stress: Analysis of mRNA expression in lymphocytes mediating panting in Jersey cattle under heat stress. *BMC Vet. Res.* **17**, 209. 10.1007/s12192-020-01076-2 (2021). 34098948 10.1186/s12917-021-02912-y PMC8186226

[45] Nikpay, M. Genome-wide search identified DNA methylation sites that regulate the metabolome. *Front. Genet.* **14**, 1093882. 10.3389/fgene.2023.1093882 (2023). 37274792 10.3389/fgene.2023.1093882 PMC10233745

[46] Campos, C. C. et al. Intramammary infusion of lipopolysaccharide promotes inflammation and alters endometrial gene expression in lactating Holstein cows. *J. Dairy Sci.* **101**, 10440–10455. 10.3168/jds.2018-14393 (2018). 30172395 10.3168/jds.2018-14393

[47] Horsley, V. & Pavlath, G. K. NFAT: Ubiquitous regulator of cell differentiation and adaptation. *J. Cell. Biol.* **156**, 771–774. 10.1083/jcb.200111073 (2002). 11877454 10.1083/jcb.200111073 PMC2173310

[48] Huang, T. et al. Nuclear factor of activated T cells (NFAT) proteins repress canonical Wnt signaling via its interaction with disheveled (Dvl) protein and participate in regulating neural progenitor cell proliferation and differentiation. *J. Biol. Chem.* **286**, 37399–37405. 10.1074/jbc.M111.251165 (2011). 21880741 10.1074/jbc.M111.251165 PMC3199487

[49] Zhang, J. et al. Genome-wide DNA methylation profile in jejunum reveals the potential genes associated with Paratuberculosis in dairy cattle. *Front. Genet.* **12**, 735147. 10.3389/fgene.2021.735147 (2021). 34721525 10.3389/fgene.2021.735147 PMC8554095

[50] Brügemann, K., Gernand, E., König von Borstel, U. & König, S. Defining and evaluating heat stress thresholds in different dairy cow production systems. *Arch. Tierzucht* **55**, 13–24 (2012).

[51] National Research Council (NRC) (U.S.). Committee on physiological effects of environmental factors on animals. In: *A guide to environmental research on animals*, 374. National Academy of Sciences: Washington, DC, USA (1971).

[52] Halli, K., Cohrs, I., Brügemann, K., Koch, C. & König, S. A pilot study on across-generation impacts of maternal heat stress on blood metabolites of female Holstein dairy calves. *Metabolites* **13**, 494. 10.3390/metabo13040494 (2023). 37110153 10.3390/metabo13040494 PMC10141042

[53] Martin, M. Cutadapt removes adapter sequences from high-throughput sequencing reads. *EMBnet J.* **17**, 10–12. 10.14806/ej.17.1.200 (2011).

[54] McKenna, A. et al. The genome analysis toolkit: a MapReduce framework for analyzing next-generation DNA sequencing data. *Genome Res.* **20**, 1297–1303. 10.1101/gr.107524.110 (2010). 20644199 10.1101/gr.107524.110 PMC2928508

[55] Yang, J. et al. Common SNPs explain a large proportion of the heritability for human height. *Nat. Genet.* **42**, 565–569. 10.1038/ng.608 (2010). 20562875 10.1038/ng.608 PMC3232052

[56] Yang, J., Lee, S. H., Goddard, M. E. & Visscher, P. M. GCTA: A tool for genome-wide complex trait analysis. *Am. J. Hum. Gent.* **88**, 76–82. 10.1016/j.ajhg.2010.11.011 (2011). 10.1016/j.ajhg.2010.11.011 PMC3014363 21167468

[57] Akalin, A. et al. methylKit: A comprehensive R package for the analysis of genome-wide DNA methylation profiles. *Genome Biol.* **13**, R87. 10.1186/gb-2012-13-10-r87 (2012). 23034086 10.1186/gb-2012-13-10-r87 PMC3491415

[58] Lawrence, M. et al. Software for computing and annotating genomic ranges. *PLoS Comput. Boil.* **9**, e1003118. 10.1371/journal.pcbi.1003118 (2013). 10.1371/journal.pcbi.1003118 PMC3738458 23950696

[59] Andrews, S. FastQC: A quality control tool for high throughput sequence data (2010).

[60] Dobin, A. et al. STAR: Ultrafast universal RNA-seq aligner. *Bioinformatics* **29**, 15–21. 10.1093/bioinformatics/bts635 (2013). 23104886 10.1093/bioinformatics/bts635 PMC3530905

[61] Love, M. I., Huber, W. & Anders, S. Moderated estimation of fold change and dispersion for RNA-seq data with DESeq2. *Genome Biol.* **15**, 550. 10.1186/s13059-014-0550-8 (2014). 25516281 10.1186/s13059-014-0550-8 PMC4302049

[62] hbctraining. DGE_workshop: Differential expression analysis with DESeq2: model fitting and hypothesis testing. GitHub Pages. https://hbctraining.github.io/DGE_workshop/lessons/05_DGE_DESeq2_analysis2.html (2024).

[63] Thomas, P. D. et al. PANTHER: Making genome-scale phylogenetics accessible to all. *Prot. Sci.* **31**, 8–22. 10.1002/pro.4218 (2022). 10.1002/pro.4218 PMC8740835 34717010

[64] Yu, G., Wang, L.-G., Han, Y. & He, Q.-Y. clusterProfiler: An R package for comparing biological themes among gene clusters. *Omics* **16**, 284–287. 10.1089/omi.2011.0118 (2012). 22455463 10.1089/omi.2011.0118 PMC3339379

[65] Yu, G. enrichplot: Visualization of Functional Enrichment Result. R package: https://www.bioconductor.org/packages/release/bioc/html/enrichplot.html and https://yulab-smu.top/biomedical-knowledge-mining-book. (2022).

[66] Singh, A. et al. DIABLO: An integrative approach for identifying key molecular drivers from multi-omics assays. *Bioinformatics* **35**, 3055–3062. 10.1093/bioinformatics/bty1054 (2019). 30657866 10.1093/bioinformatics/bty1054 PMC6735831

[67] Rohart, F., Gautier, B., Singh, A. & Lê Cao, K.-A. mixOmics: An R package for ‘omics feature selection and multiple data integration. *PLoS Comput. Boil.* **13**, e1005752. 10.1371/journal.pcbi.1005752 (2017). 10.1371/journal.pcbi.1005752 PMC5687754 29099853

