# Supplementary figures and images for: Heat stress induces specific methylation, transcriptomic and metabolic pattern in dairy cows and their female progeny
Source: Sci Rep. 2025 May 16;15:17021. doi: 10.1038/s41598-025-01082-3 (PMC12084553; doi:10.1038/s41598-025-01082-3)

First components

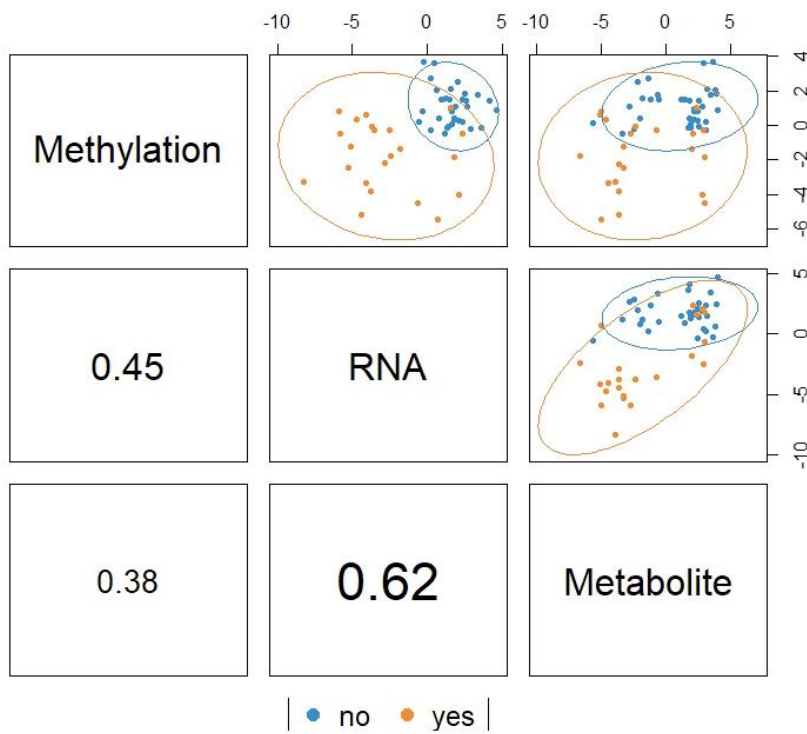

Second components

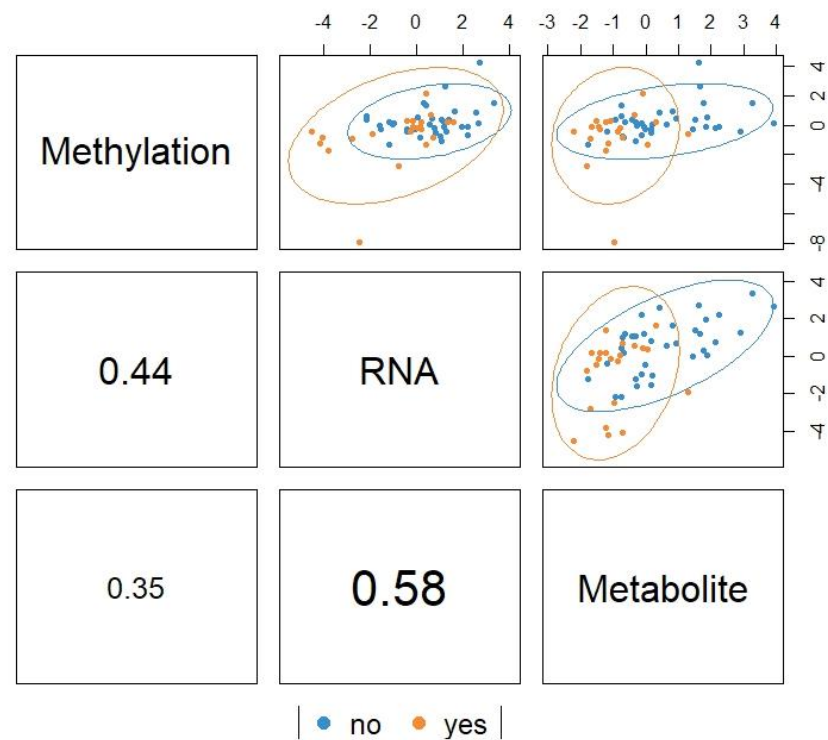

Supplement: Supplementary file 1 — Supplementary Information 1. [file 41598_2025_1082_MOESM1_ESM.pdf]

## Components 1 and 2

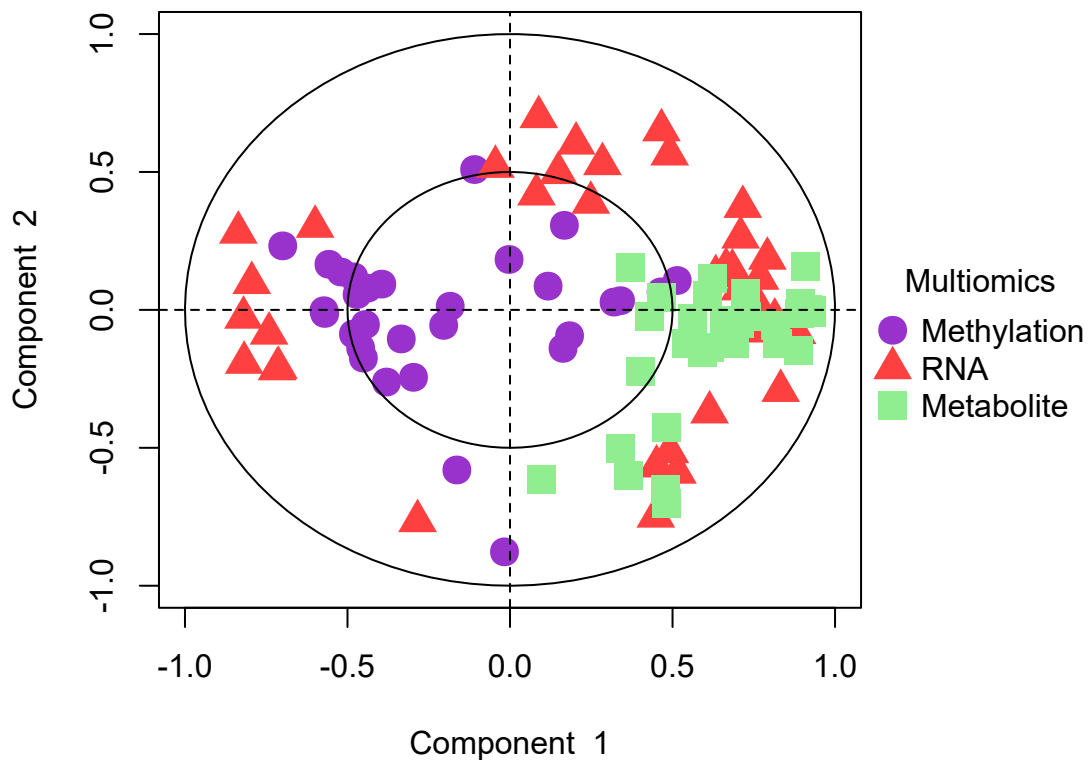

Supplement: Supplementary file 3 — Supplementary Information 3. [file 41598_2025_1082_MOESM3_ESM.pdf]
