# Supplementary material for: Heat stress induces specific methylation, transcriptomic and metabolic pattern in dairy cows and their female progeny
Source: Sci Rep. 2025 May 16;15:17021. doi: 10.1038/s41598-025-01082-3 (PMC12084553; doi:10.1038/s41598-025-01082-3)
Supplement: Supplementary file 2 — Supplementary Information 2. [file 41598_2025_1082_MOESM2_ESM.pdf]

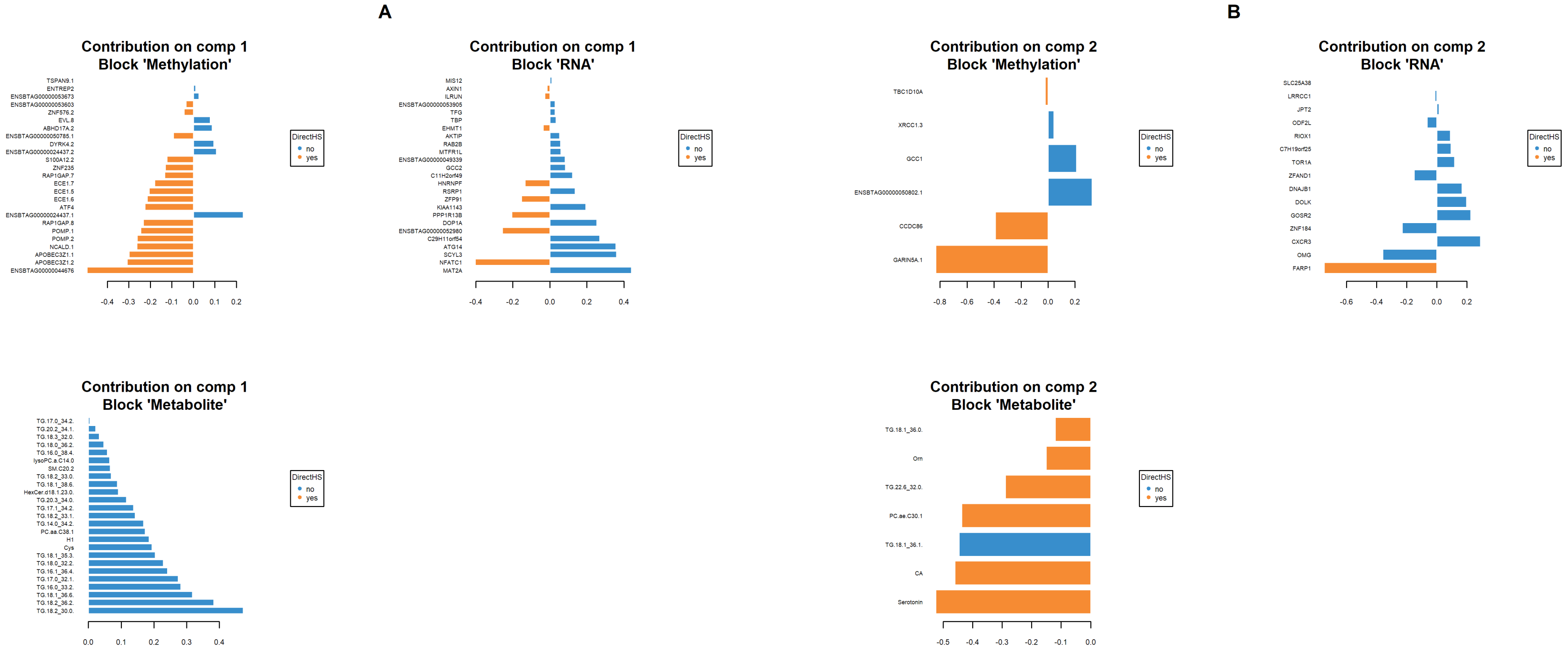
A
B
Contribution on comp 1
Block 'Methylation'
TSPAN9.1
ENTREP2
ENSBTAG00000053673
ENSBTAG00000053603
ZNF576.2
EVL.8
ABHD17A.2
ENSBTAG00000050785.1
DYRK4.2
ENSBTAG00000024437.2
S100A12.2
ZNF235
RAP1GAP.7
ECE1.7
ECE1.5
ECE1.6
ATF4
ENSBTAG00000024437.1
RAP1GAP.8
POMP.1
POMP.2
NCALD.1
APOBEC3Z1.1
APOBEC3Z1.2
ENSBTAG00000044676
-0.4 -0.3 -0.2 -0.1 0.0 0.1 0.2
DirectHS
no
yes
Contribution on comp 1
Block 'RNA'
MIS12
AXIN1
ILRUN
ENSBTAG00000053905
TFG
TBP
EHMT1
AKTIP
RAB2B
MTFR1L
ENSBTAG00000049339
GCC2
C11H2orf49
HNRNPF
RSRP1
ZFP91
KIAA1143
PPP1R13B
DOP1A
ENSBTAG00000052980
C29H11orf54
ATG14
SCYL3
NFATC1
MAT2A
-0.4 -0.2 0.0 0.2 0.4
DirectHS
no
yes
Contribution on comp 2
Block 'Methylation'
TBC1D10A
XRCC1.3
GCC1
ENSBTAG00000050802.1
CCDC86
GARIN5A.1
-0.8 -0.6 -0.4 -0.2 0.0 0.2
DirectHS
no
yes
Contribution on comp 2
Block 'RNA'
SLC25A38
LRRCC1
JPT2
ODF2L
RIOX1
C7H19orf25
TOR1A
ZFAND1
DNAJB1
DOLK
GOSR2
ZNF184
CXCR3
OMG
FARP1
-0.6 -0.4 -0.2 0.0 0.2
DirectHS
no
yes
Contribution on comp 1
Block 'Metabolite'
TG.17.0_34.2.
TG.20.2_34.1.
TG.18.3_32.0.
TG.18.0_36.2.
TG.16.0_38.4.
lysoPC.a.C14.0
SM.C20.2
TG.18.2_33.0.
TG.18.1_38.6.
HexCer.d18.1.23.0.
TG.20.3_34.0.
TG.17.1_34.2.
TG.18.2_33.1.
TG.14.0_34.2.
PC.aa.C38.1
H1
Cys
TG.18.1_35.3.
TG.18.0_32.2.
TG.16.1_36.4.
TG.17.0_32.1.
TG.16.0_33.2.
TG.18.1_36.6.
TG.18.2_36.2.
TG.18.2_30.0.
0.0 0.1 0.2 0.3 0.4
DirectHS
no
yes
Contribution on comp 2
Block 'Metabolite'
TG.18.1_36.0.
Orn
TG.22.6_32.0.
PC.ae.C30.1
TG.18.1_36.1.
CA
Serotonin
-0.5 -0.4 -0.3 -0.2 -0.1 0.0
DirectHS
no
yes
