# Supplementary material for: Heat stress induces specific methylation, transcriptomic and metabolic pattern in dairy cows and their female progeny
Source: Sci Rep. 2025 May 16;15:17021. doi: 10.1038/s41598-025-01082-3 (PMC12084553; doi:10.1038/s41598-025-01082-3)
Supplement: Supplementary file 8 — Supplementary Information 8. [file 41598_2025_1082_MOESM8_ESM.pdf]

Supplementary material

Figure S1. Diagnostic plots based on the first two principal components of methylation, RNAseq and metabolite data from dams with (orange dots) and without (blue dots) direct heat stress. The values below the diagonal indicate the correlation coefficients between the first and second components from each dataset, respectively.

Figure S2. Contributions of the selected features from the different “omics- blocks” methylation, RNAseq and metabolites to principal component 1 (**A**) and principal component 2 (**B**).

Figure S3. Correlations of selected “omics-features (see Figure S1) with principal components 1 and 2.

Table S1. Genes with differently methylated promoters in heat stressed cows (direct heat stress effects) and in heat stressed calves (maternal indirect heat stress effects).

Table S2. GO enrichment with regard to biological processes in heat stressed dams and maternal heat stress in calves, as well as respective gene set enrichment analyses (GSEA).

Table S3. Overview of correlated features between gene expressions (i.e., list of the relevant genes) and metabolites with significantly different measurements in heat stressed dams compared to the control group.

Table S4. List of Metabolites: MxP® Quant 500 Kit (Biocrates Life Sciences AG, Innsbruck, Austria), Version: 2_02/2019
